# Supplementary material for: Evolution of enzyme functionality in the flavin-containing monooxygenases
Source: Nat Commun. 2023 Feb 24;14:1042. doi: 10.1038/s41467-023-36756-x (PMC9950137; doi:10.1038/s41467-023-36756-x)
Supplement: Supplementary file 1 — Supplementary information [file 41467_2023_36756_MOESM1_ESM.pdf]

Evolution of enzyme functionality in the flavin-containing monooxygenases

Gautier Bailleul[1], Guang Yang[1], Callum R. Nicoll[2], Andrea Mattevi[2], Marco W. Fraaije[1], Maria Laura Mascotti[1,3*]

[1] Molecular Enzymology group, University of Groningen; Groningen, The Netherlands.

[2] Department of Biology and Biotechnology "Lazzaro Spallanzani", University of Pavia; Pavia, Italy.

[3] IMIBIO-SL CONICET, Facultad de Química Bioquímica y Farmacia, Universidad Nacional de San Luis; San Luis, Argentina.

[*]Corresponding author. Email: m.l.mascotti@rug.nl.

These authors contributed equally: Gautier Bailleul, Guang Yang.

SUPPLEMENTARY INFORMATION

List of contents

Supplementary Fig. 1

Supplementary Fig. 2

Supplementary Fig. 3

Supplementary Fig. 4

Supplementary Table 1

Supplementary Fig. 5

Supplementary Fig. 6

Supplementary Table 2

Supplementary Fig. 7

Supplementary Fig. 8

Supplementary Fig. 9

Supplementary Fig. 10

Supplementary Table 3

Supplementary Table 4

Supplementary Table 5

Supplementary Fig. 11

Supplementary Fig. 12

Supplementary Fig. 13

Supplementary Fig. 14

Supplementary Fig. 15

Supplementary Fig. 16
Supplementary Fig. 17
Supplementary References

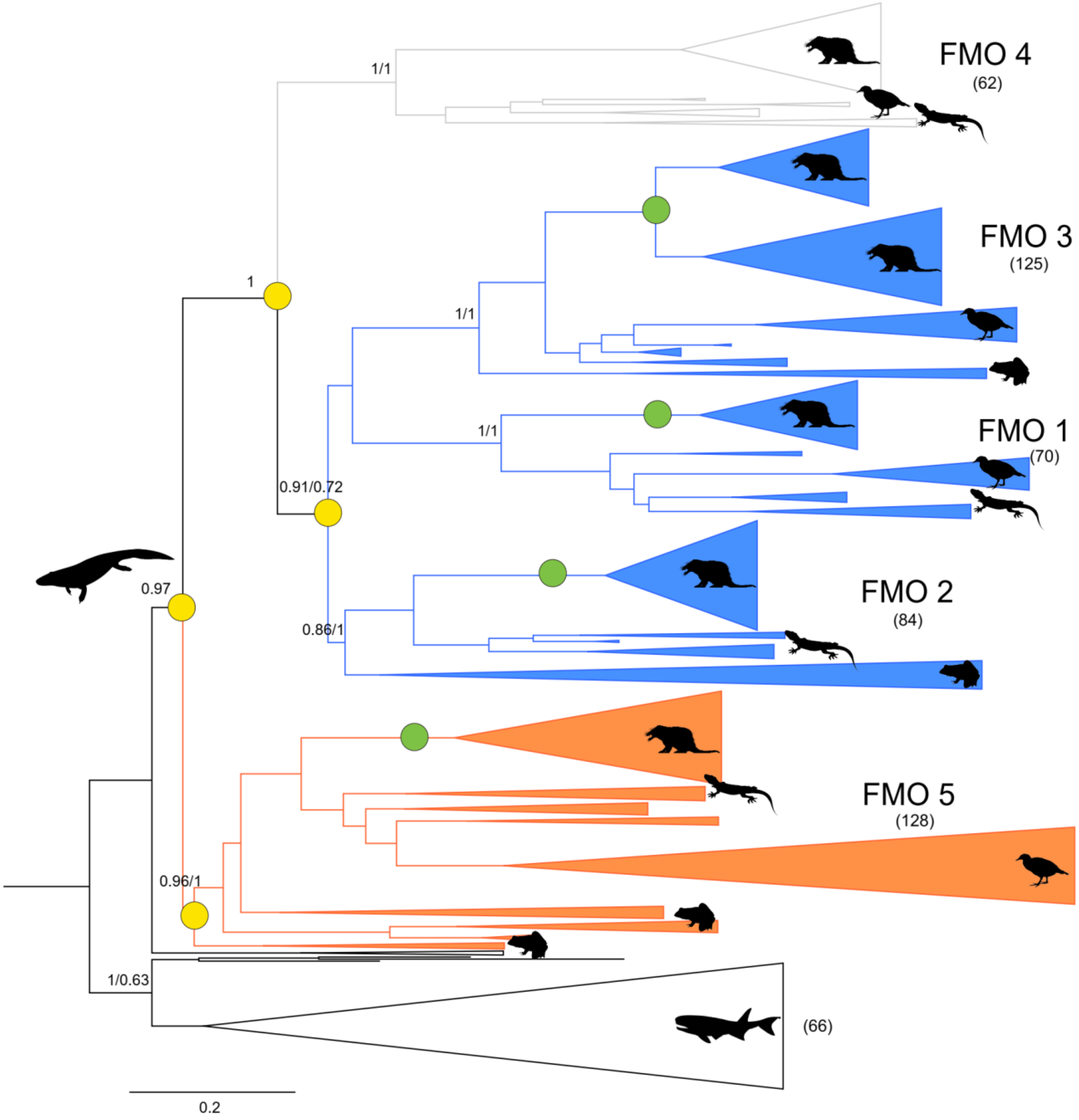

Supplementary Fig. 1

Vertebrates' phylogeny of FMOs with resurrected nodes marked. The tree was constructed in RAxML v8.2.10, 500 non-parametric bootstraps were run and best-fit model parameters were obtained with ProtTest v3.4. Bootstrap transfer was applied to obtain TBE support values. Bayesian inference was also performed in Mr. Bayes 3.2.6 using the same MSA and therefore support values (TBE/PP) are shown at the nodes corresponding to major divergences. The scale bar represents the substitutions per site. The employed MSA was trimmed at single sequence extensions and contained 536 taxa and 537 sites. Clades are collapsed and colored according to the inferred functionality, S/N oxidation in blue and BV oxidation in orange. FMO 4

clade is colored in grey as no experimental data confirming activity is available. Below the clade names the number of contained sequences is given in brackets. Actinopterygii, coelacanthimorpha and chondrichthyes sequences populate the root and are shown in black branches. The four ancestral nodes experimentally characterized in this work are represented with yellow circles and the mammalian ancestors previously characterized by us[1,2] are shown with green circles. The depicted silhouettes (obtained from http://www.phylopic.org/) represent the ancestral tetrapod (by Dmitry Bogdanov (vectorized by T. Michael Keesey) under CC BY-SA 3.0) and the extant mammals, aves, amphibia (by Nobu Tamura and T. Michael Keesey under CC BY-SA 3.0), lepidosauria (by Ghedo and T. Michael Keesey under CC BY-SA 3.0) and actinopterygii (ray-finned fish).

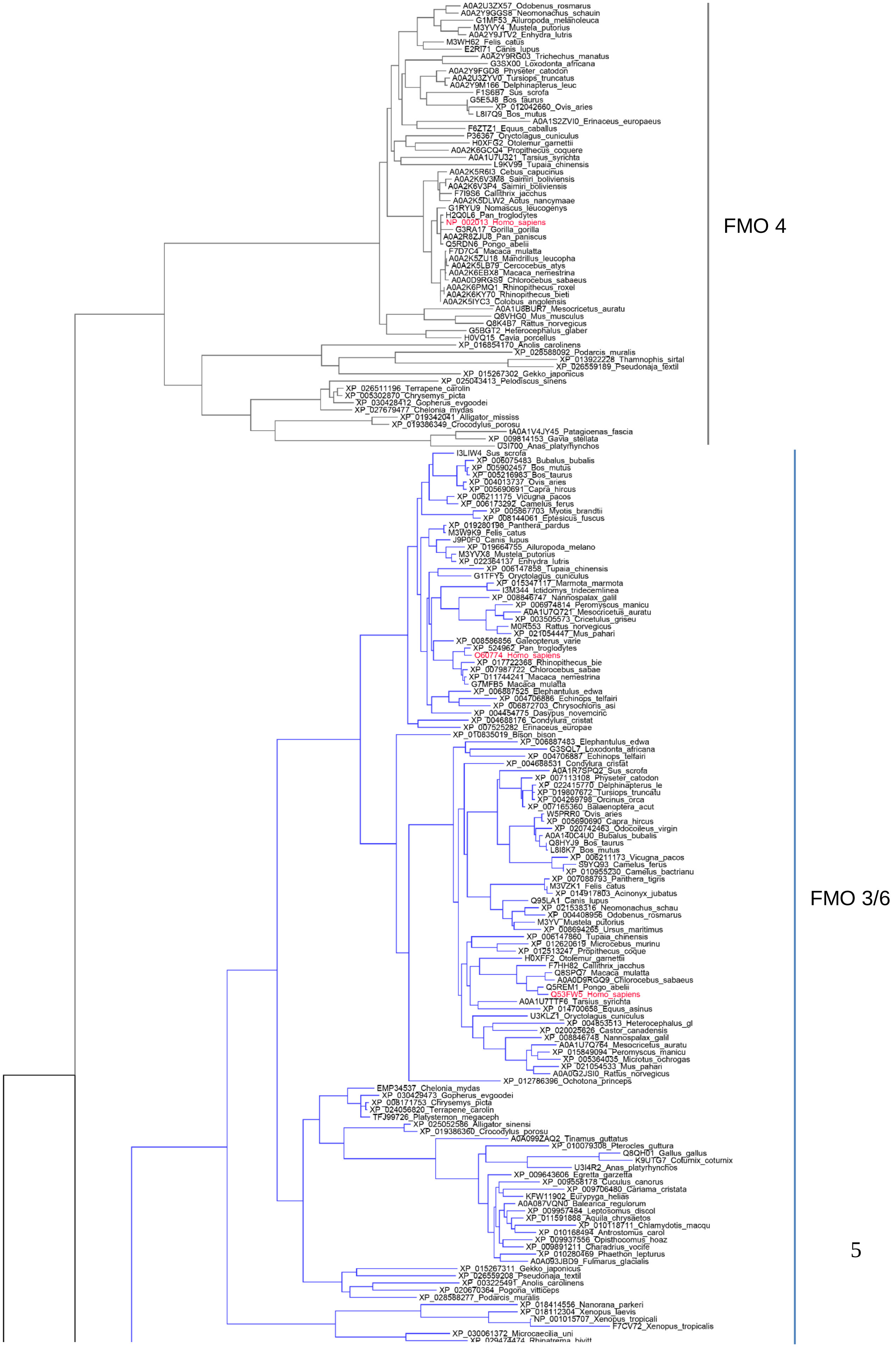
FMO 4
FMO 3/6

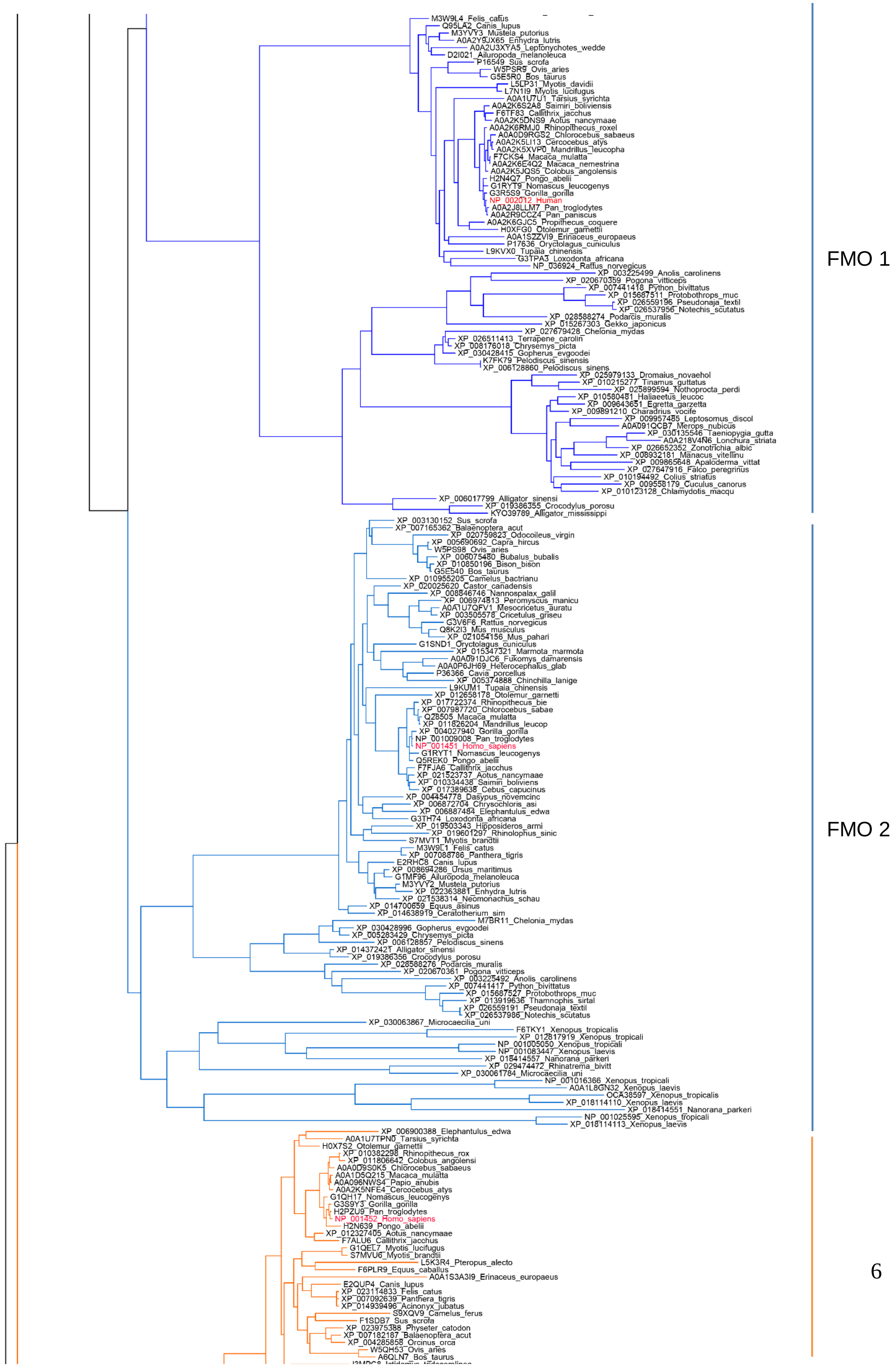
M3W9L4_Felis_catus
Q95LA2_Canis_lupus
M3YVY3_Mustela_putorius
A0A2Y9JX65_Enhydra_lutris
A0A2U3XYA5_Leptonychotes_wedde
D2I021_Ailuropoda_melanoleuca
P16549_Sus_scrofa
W5PSR9_Ovis_aries
G5E5R0_Bos_taurus
L5LP31_Myotis_davidii
L7N1I9_Myotis_lucifugus
A0A1U7U1_Tarsius_syrichta
A0A2K6S2A8_Saimiri_boliviensis
F6TF83_Callithrix_jacchus
A0A2K5DNS9_Aotus_nancymaae
A0A2K6RMJ0_Rhinopithecus_roxel
A0A0D9RGS2_Chlorocebus_sabaeus
A0A2K5LI13_Cercocebus_atys
A0A2K5XVP0_Mandrillus_leucopha
F7CKS4_Macaca_mulatta
A0A2K6E4Q2_Macaca_nemestrina
A0A2K5JQS5_Colobus_angolensis
H2N4Q7_Pongo_abelii
G1RYT9_Nomascus_leucogenys
G3R5S9_Gorilla_gorilla
NP_002012_Human
A0A2J8LLM7_Pan_troglodytes
A0A2R9CCZ4_Pan_paniscus
A0A2K6GJC5_Propithecus_coquere
H0XFG0_Otolemur_garnettii
A0A1S2ZVI9_Erinaceus_europaeus
P17636_Oryctolagus_cuniculus
L9KVX0_Tupaia_chinensis
G3TPA3_Loxodonta_africana
NP_036924_Rattus_norvegicus
XP_003225499_Anolis_carolinens
XP_020670359_Pogona_vitticeps
XP_007441418_Python_bivittatus
XP_015687511_Protobothrops_muc
XP_026559196_Pseudonaja_textil
XP_026537956_Notechis_scutatus
XP_028588274_Podarcis_muralis
XP_015267303_Gekko_japonicus
XP_027679428_Chelonia_mydas
XP_026511413_Terrapene_carolin
XP_008176018_Chrysemys_picta
XP_030428415_Gopherus_evgoodei
K7FK79_Pelodiscus_sinensis
XP_006T28860_Pelodiscus_sinens
XP_025979133_Dromaius_novaehol
XP_010215277_Tinamus_guttatus
XP_025899594_Nothoprocta_perdi
XP_010580481_Haliaeetus_leucoc
XP_009643651_Egretta_garzetta
XP_009891210_Charadrius_vocife
XP_009957485_Leptosomus_discol
A0A091QCB7_Merops_nubicus
XP_030135546_Taeniopygia_gutta
A0A218V4N6_Lonchura_striata
XP_026652352_Zonotrichia_albic
XP_008932181_Manacus_vitellinu
XP_009865648_Apaloderma_vittat
XP_027647916_Falco_peregrinus
XP_010194492_Colius_striatus
XP_009558179_Cuculus_canorus
XP_010123128_Chlamydotis_macqu
XP_006017799_Alligator_sinensi
XP_019386355_Crocodylus_porosu
KYO39789_Alligator_mississippi
FMO 1
XP_003130152_Sus_scrofa
XP_007165362_Balaenoptera_acut
XP_020759823_Odocoileus_virgin
XP_005690692_Capra_hircus
W5PS98_Ovis_aries
XP_006075480_Bubalus_bubalis
XP_010850196_Bison_bison
G5E540_Bos_taurus
XP_010955205_Camelus_bactrianu
XP_020025620_Castor_canadensis
XP_008846746_Nannospalax_galil
XP_006974813_Peromyscus_manicu
A0A1U7QFV1_Mesocricetus_auratu
XP_003505578_Cricetulus_griseu
G3V6F6_Rattus_norvegicus
Q8K2I3_Mus_musculus
XP_021054156_Mus_pahari
G1SND1_Oryctolagus_cuniculus
XP_015347321_Marmota_marmota
A0A091DJC6_Fukomys_damarensis
A0A0P6JH69_Heterocephalus_glab
P36366_Cavia_porcellus
XP_005374888_Chinchilla_lanige
L9KUM1_Tupaia_chinensis
XP_012658178_Otolemur_garnetti
XP_017722374_Rhinopithecus_bie
XP_007987720_Chlorocebus_sabae
Q28505_Macaca_mulatta
XP_011826204_Mandrillus_leucop
XP_004027940_Gorilla_gorilla
NP_001009008_Pan_troglodytes
NP_001451_Homo_sapiens
G1RYT1_Nomascus_leucogenys
Q5REK0_Pongo_abelii
F7FJA6_Callithrix_jacchus
XP_021523737_Aotus_nancymaae
XP_010334438_Saimiri_boliviens
XP_017389638_Cebus_capucinus
XP_004454778_Dasypus_novemcinc
XP_006872704_Chrysochloris_asi
XP_006887484_Elephantulus_edwa
G3TH74_Loxodonta_africana
XP_019503343_Hipposideros_armi
XP_019601297_Rhinolophus_sinic
S7MVT1_Myotis_brandtii
M3W9L1_Felis_catus
XP_007088786_Panthera_tigris
E2RHC8_Canis_lupus
XP_008694286_Ursus_maritimus
G1MF96_Ailuropoda_melanoleuca
M3YVY2_Mustela_putorius
XP_022363881_Enhydra_lutris
XP_021538314_Neomonachus_schau
XP_014700659_Equus_asinus
XP_014638919_Ceratotherium_sim
M7BR11_Chelonia_mydas
XP_030428996_Gopherus_evgoodei
XP_005283429_Chrysemys_picta
XP_006128857_Pelodiscus_sinens
XP_014372427_Alligator_sinensi
XP_019386356_Crocodylus_porosu
XP_028588276_Podarcis_muralis
XP_020670361_Pogona_vitticeps
XP_003225492_Anolis_carolinens
XP_007441417_Python_bivittatus
XP_015687527_Protobothrops_muc
XP_013919636_Thamnophis_sirtal
XP_026559191_Pseudonaja_textil
XP_026537986_Notechis_scutatus
XP_030063867_Microcaecilia_uni
F6TKY1_Xenopus_tropicalis
XP_012817919_Xenopus_tropicali
NP_001005050_Xenopus_tropicali
NP_001083447_Xenopus_laevis
XP_018414557_Nanorana_parkeri
XP_029474472_Rhinatrema_bivitt
XP_030061784_Microcaecilia_uni
NP_001016366_Xenopus_tropicali
A0A1L8GN32_Xenopus_laevis
OCA38597_Xenopus_tropicalis
XP_018114110_Xenopus_laevis
XP_018414551_Nanorana_parkeri
NP_001025595_Xenopus_tropicali
XP_018114113_Xenopus_laevis
FMO 2
XP_006900388_Elephantulus_edwa
A0A1U7TPN0_Tarsius_syrichta
H0X7S2_Otolemur_garnettii
XP_010382298_Rhinopithecus_rox
XP_011806642_Colobus_angolensi
A0A0D9S0K5_Chlorocebus_sabaeus
A0A1D5Q215_Macaca_mulatta
A0A096NWS4_Papio_anubis
A0A2K5NFE4_Cercocebus_atys
G1QH17_Nomascus_leucogenys
G3S9Y3_Gorilla_gorilla
H2PZU9_Pan_troglodytes
NP_001452_Homo_sapiens
H2N639_Pongo_abelii
XP_012327405_Aotus_nancymaae
F7ALU6_Callithrix_jacchus
G1QEL7_Myotis_lucifugus
S7MVU6_Myotis_brandtii
L5K3R4_Pteropus_alecto
F6PLR9_Equus_caballus
A0A1S3A3I9_Erinaceus_europaeus
E2QUP4_Canis_lupus
XP_023114833_Felis_catus
XP_007092639_Panthera_tigris
XP_014939496_Acinonyx_jubatus
S9XQV9_Camelus_ferus
F1SDB7_Sus_scrofa
XP_023975388_Physeter_catodon
XP_007182187_Balaenoptera_acut
XP_004285858_Orcinus_orca
W5QH53_Ovis_aries
A6QLN7_Bos_taurus

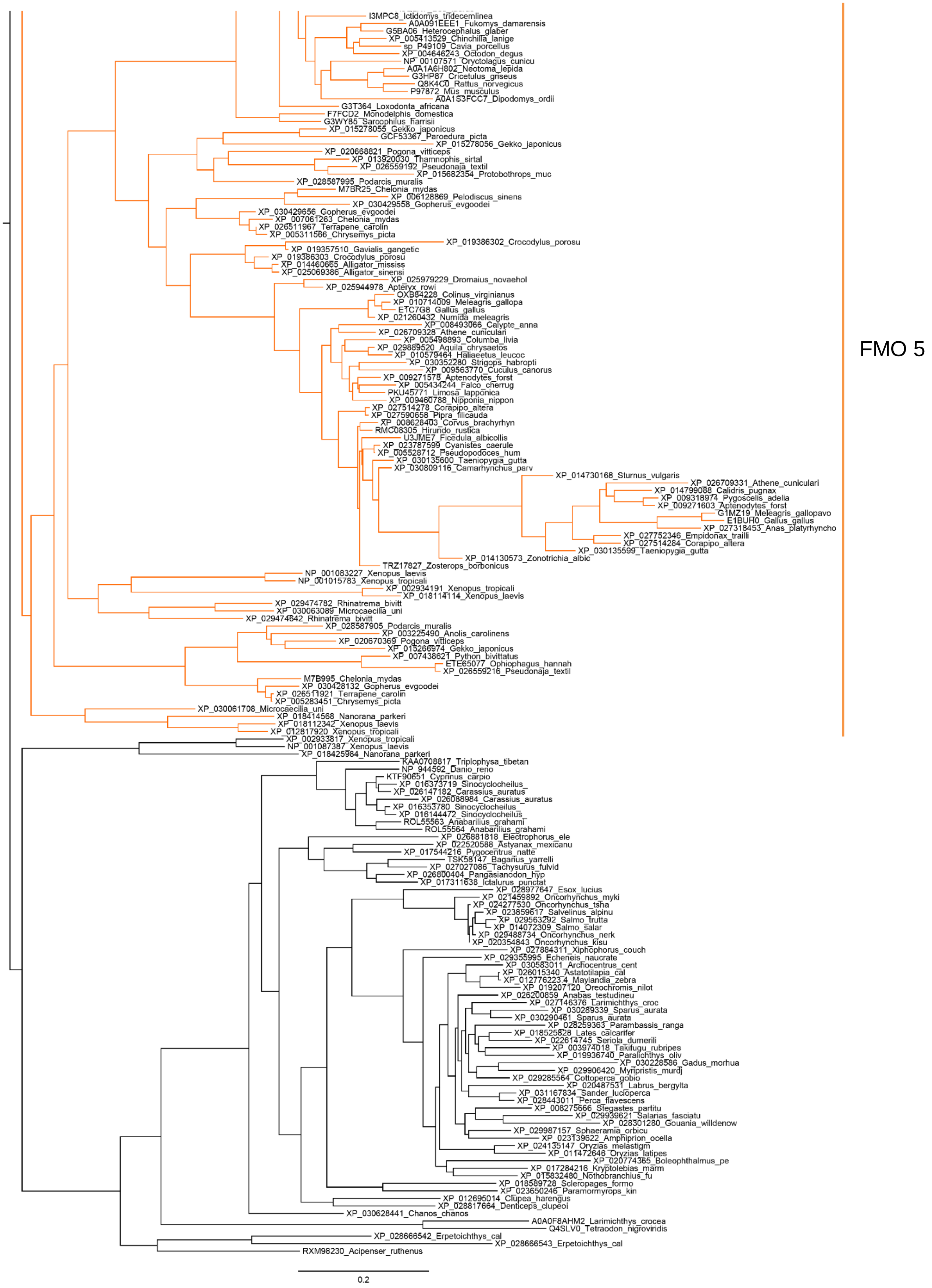
I3MPC8_Ictidomys_tridecemlinea
A0A091EEE1_Fukomys_damarensis
G5BA06_Heterocephalus_glaber
XP_005413529_Chinchilla_lanige
sp_P49109_Cavia_porcellus
XP_004646243_Octodon_degus
NP_001075711_Oryctolagus_cunicu
A0A1A6H802_Neotoma_lepida
G3HP87_Cricetulus_griseus
Q8K4C0_Rattus_norvegicus
P97872_Mus_musculus
A0A1S3FCC7_Dipodomys_ordii
G3T364_Loxodonta_africana
F7FCD2_Monodelphis_domestica
G3WY85_Sarcophilus_harrisii
XP_015278055_Gekko_japonicus
GCF53367_Paroedura_picta
XP_015278056_Gekko_japonicus
XP_020668821_Pogona_vitticeps
XP_013920030_Thamnophis_sirtal
XP_026559192_Pseudonaja_textil
XP_015682354_Protobothrops_muc
XP_028587995_Podarcis_muralis
M7BR25_Chelonia_mydas
XP_006128869_Pelodiscus_sinens
XP_030429558_Gopherus_evgoodei
XP_030429656_Gopherus_evgoodei
XP_007061263_Chelonia_mydas
XP_026511967_Terrapene_carolin
XP_005311566_Chrysemys_picta
XP_019386302_Crocodylus_porosu
XP_019357510_Gavialis_gangetic
XP_019386303_Crocodylus_porosu
XP_014460665_Alligator_mississ
XP_025069386_Alligator_sinensi
XP_025979229_Dromaius_novaehol
XP_025944978_Apteryx_rowi
OXB64228_Colinus_virginianus
XP_010714009_Meleagris_gallopa
ETC7G8_Gallus_gallus
XP_021260432_Numida_meleagris
XP_008493066_Calypte_anna
XP_026709328_Athene_cunicular
XP_005498893_Columba_livia
XP_029889520_Aquila_chrysaetos
XP_010579464_Haliaeetus_leucoc
XP_030352280_Strigops_habropti
XP_009563770_Cuculus_canorus
XP_009271578_Aptenodytes_forst
XP_005434244_Falco_cherrug
PKU45771_Limosa_lapponica
XP_009460788_Nipponia_nippon
XP_027514278_Corapipo_altera
XP_027590658_Pipra_filicauda
XP_008628403_Corvus_brachyrhyn
RMC08305_Hirundo_rustica
U3JME7_Ficedula_albicollis
XP_023787599_Cyanistes_caerule
XP_005528712_Pseudopodoces_hum
XP_030135600_Taeniopygia_gutta
XP_030809116_Camarhynchus_parv
XP_014730168_Sturnus_vulgaris
XP_026709331_Athene_cunicularI
XP_014799088_Calidris_pugnax
XP_009318974_Pygoscelis_adelia
XP_009271603_Aptenodytes_forst
G1MZ19_Meleagris_gallopavo
E1BUH0_Gallus_gallus
XP_027318453_Anas_platyrhyncho
XP_027752346_Empidonax_traillii
XP_027514284_Corapipo_altera
XP_030135599_Taeniopygia_gutta
XP_014130573_Zonotrichia_albic
TRZ17827_Zosterops_borbonicus
NP_001083227_Xenopus_laevis
NP_001015783_Xenopus_tropicali
XP_002934191_Xenopus_tropicali
XP_018114114_Xenopus_laevis
XP_029474782_Rhinatrema_bivitt
XP_030063089_Microcaecilia_uni
XP_029474642_Rhinatrema_bivitt
XP_028587905_Podarcis_muralis
XP_003225490_Anolis_carolinens
XP_020670369_Pogona_vitticeps
XP_015266974_Gekko_japonicus
XP_007438621_Python_bivittatus
ETE65077_Ophiophagus_hannah
XP_026559216_Pseudonaja_textil
M7B995_Chelonia_mydas
XP_030428132_Gopherus_evgoodei
XP_026511921_Terrapene_carolin
XP_005283451_Chrysemys_picta
XP_030061708_Microcaecilia_uni
XP_018414568_Nanorana_parkeri
XP_018112342_Xenopus_laevis
XP_012817920_Xenopus_tropicali
XP_002933817_Xenopus_tropicali
NP_001087387_Xenopus_laevis
XP_018425984_Nanorana_parkeri
KAA0708817_Triplophysa_tibetan
NP_944592_Danio_rerio
KTF90651_Cyprinus_carpio
XP_016373719_Sinocyclocheilus_
XP_026147182_Carassius_auratus
XP_026088984_Carassius_auratus
XP_016353780_Sinocyclocheilus_
XP_016144472_Sinocyclocheilus_
ROL55563_Anabarilius_grahami
ROL55564_Anabarilius_grahami
XP_026881818_Electrophorus_ele
XP_022520588_Astyanax_mexicanu
XP_017544216_Pygocentrus_natte
TSK58147_Bagarius_yarrelli
XP_027027086_Tachysurus_fulvid
XP_026800404_Pangasianodon_hyp
XP_017311638_Ictalurus_punctat
XP_028977647_Esox_lucius
XP_021459892_Oncorhynchus_myki
XP_024277530_Oncorhynchus_tsha
XP_023859617_Salvelinus_alpinu
XP_029563292_Salmo_trutta
XP_014072309_Salmo_salar
XP_029488734_Oncorhynchus_nerk
XP_020354843_Oncorhynchus_kisu
XP_027884311_Xiphophorus_couch
XP_029355995_Echeneis_naucrate
XP_030583011_Archocentrus_cent
XP_026015340_Astatotilapia_cal
XP_012776223_4_Maylandia_zebra
XP_019207120_Oreochromis_nilot
XP_026200859_Anabas_testudineu
XP_027146376_Larimichthys_croc
XP_030289339_Sparus_aurata
XP_030290461_Sparus_aurata
XP_028259363_Parambassis_ranga
XP_018525828_Lates_calcarifer
XP_022614745_Seriola_dumerili
XP_003974018_Takifugu_rubripes
XP_019936740_Paralichthys_oliv
XP_030228586_Gadus_morhua
XP_029906420_Myripristis_murdj
XP_029285564_Cottoperca_gobio
XP_020487531_Labrus_bergylta
XP_031167834_Sander_lucioperca
XP_028443011_Perca_flavescens
XP_008275666_Stegastes_partitu
XP_029939621_Salarias_fasciatu
XP_028301280_Gouania_willdenow
XP_029987157_Sphaeramia_orbicu
XP_023139622_Amphiprion_ocella
XP_024135147_Oryzias_melastigm
XP_011472646_Oryzias_latipes
XP_020774365_Boleophthalmus_pe
XP_017284216_Kryptolebias_marm
XP_015832480_Nothobranchius_fu
XP_018589728_Scleropages_formo
XP_023650246_Paramormyrops_kin
XP_012695014_Clupea_harengus
XP_028817664_Denticeps_clupeoi
XP_030628441_Chanos_chanos
A0A0F8AHM2_Larimichthys_crocea
Q4SLV0_Tetraodon_nigroviridis
XP_028666542_Erpetoichthys_cal
XP_028666543_Erpetoichthys_cal
RXM98230_Acipenser_ruthenus
FMO 5
0.2

Supplementary Fig. 2

Fully annotated vertebrates' phylogeny of FMOs. The tree from Supplementary Fig. 1 is presented showing at the tip of the branches the accession codes and species names for all included sequences in the phylogeny. Clades are colored according to functionality as throughout the article. As a reference, *Homo sapiens* sequences are shown in red. The scale bar represents the substitutions per site.

>tAncFMO1-5
MAKRVAVIGAGASGLTAIKCCLDEGLEPTCFERSDDIGGLWRFKENPEDGRASIYKSVIINTSKEMMCYSDFPIPDD
FPNYMHNSKIMEYFRMYAKHFDLLKYIRFKTTVCSVKKRPDFSTTGQWDVVTETDGKQESAIFDAVLVCTGHHTDPH
LPLDSFPGIEKFKGQYFHSRDYKNPEEFQGKRVLVIGIGNSGGDIAVELSRTAKQVFLSTRRGSWILNRVSDNGYPL
DMVHSTRFKNLLKHILPSSLVNWMAEKKMNQRFNHENYGLKPQHRIFSQHPMVNDDLPNRIISGTVLVKPNVKEFTE
TSAIFEDGTVEENIDVVIFATGYSFSFPFLEESVLKVQNNKVSLYKYVFPPHLEKPTLAVIGLIQPLGAIMPISELQ
ARWATRVFKGLNKLPSANTMMADIAKKKEEMEKRYVTSQRHTIQVDYIEYMDELASLIGVKPNLLSLFLTDPKLALE
VFFGPCTPYQYRLTGPGKWDGARKAILTQWDRIIKPTKTRVLQDNSSSSSMPLLLKILGLALLSALYYFL

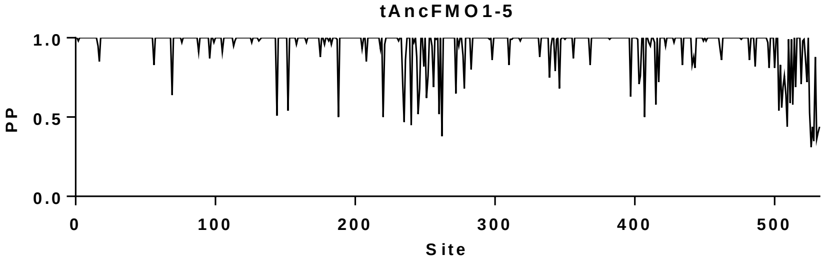

>tAncFMO5
MAKRVAVIGAGSSGLTAIKCCLDEGLEPTCFERSDDIGGLWRFKENPEDGRASIYKSVIINTSKEMMCFSDFPIPDD
FPNYMHNSKIMEYFRMYAKHFDLLKYIRFKTTVCSVKKRPDFSTTGQWDVVTETDGKQESAIFDAVLVCTGHHTNPH
LPLDSFPGIEKFKGQYFHSRDYKNPQEFQGKRVIVIGIGNSGGDLAVELSHTAKQVFLSTRRGAWILNRVSDNGYPL
DVVLSTRFKNLLKQILPTSMVNRWAEKKMNARFNHENYGLKPQHRILSQHPTVNDDLPNRIISGKVLVKPNVKEFTE
TAAIFEDGTVEENIDVVIFATGYSFSFPFLEDSVLKVQNNKVSLYKFVFPPHLEKPTLAIIGLIQPLGAIMPISELQ
ARWATRVFKGLNKLPSANDMMADIAKKKEEMEKRYVTSQRHTIQVDYIEYMDELASLIGVKPNLLSLFLTDPKLAWE
VFFGPCTPYQYRLTGPGKWDGARKAILTQRDRIIKPTKTRVLQDNNTSSSMPLLLKILGLALLSALYYFL

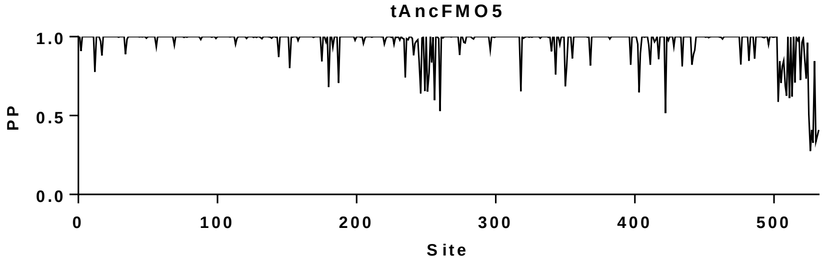

>tAncFMO1-4
MAKRVAVIGAGVSGLTSIKCCLDEGLEPTCFERSDDIGGLWRFTENPEDGRASIYKSVITNTSKEMMCYSDFPFPED
FPNYMHNSKFLEYLRMYAKHFDLLKYIQFKTTVCSVKKRPDFSTTGQWDVVTETDGKQESAIFDAVMVCTGHHTDPH
LPLDSFPGIEKFKGQYFHSREYKNPEEFQGKRVLVIGIGNSGGDIAVELSRTAKQVFLSTRRGSWVLSRVSDNGYPL
DMVFTTRFQNLLRNILPSSLVNWMTEKKMNQWFNHENYGLVPQNRILSQHPVVNDDLPSRIICGTVVVKPNVKEFTE
TSAIFEDGTVEENIDVVIFATGYSFSFPFLEESVIKVENNKVSLYKYVFPPHLEKPTLAVIGLIQPLGAIMPTAELQ
ARWATRVFKGLCKLPSANTMMADIAKKKEKMIKRFVTSQRNTIQTDYIEYMDELASFIGVKPNILSLFLTDPKLALE
VFFGPCTPYQYRLTGPGKWDGARNAILTQWDRILKPTKTRVLQDDSSSNSMPFLLKILGLALLSALYYFL

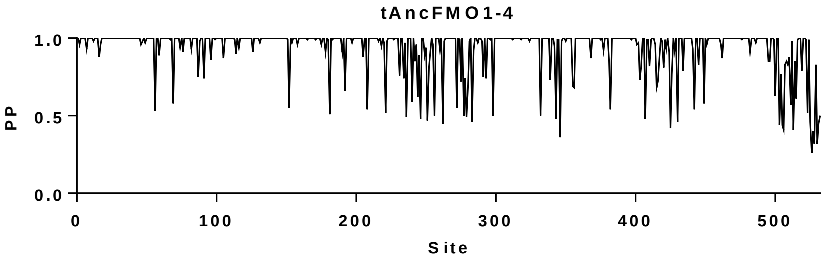

```
>tAncFMO1-3
MAKRVAVIGAGVSGLTSIKCCLDEGLEPTCFERSDDIGGLWRFTENVEDGRASIYKSVITNTSKEMMCYSDFPFPED
FPNYMHNSKFLEYLRMYAKHFDLLKYIQFKTTVCSVKKCPDFSTTGQWDVVTETDGKQESAIFDAVMVCTGHHTDPY
LPLDSFPGIEKFKGQYFHSREYKNPEGFQGKRVLVIGMGNSGADIAVELSHTAKQVFLSTRRGSWVMSRVSDNGYPW
DMVFTTRFQNLLRNVLPSSIVNWMTEKKMNQWFNHENYGLVPQNRTLMKEPVFNDDLPSRIICGTVVVKPNVKEFTE
TSAIFEDGTVEENIDVVIFATGYTFSFPFLDESVIKVENNKVSLYKYVFPPHLEKPTLAVIGLIQPLGAIMPTAELQ
ARWATRVFKGLCKLPSANTMMEDIAKKKEKKIKWFGTSQSNTLQTDYIEYMDELASFIGAKPNILSLFLTDPKLALE
VFFGPCTPYQYRLTGPGKWDGARNAILTQWDRILKPTRTRVLQNDSSSNSVPFLLKILGLALLSALYYFL
```

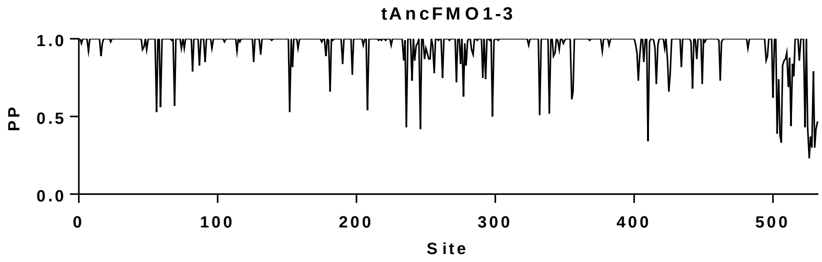

Supplementary Fig. 3

Reconstructed tAncFMOs. Fasta sequences of the reconstructed tAncFMOs selected for experimental characterization. Scatter graphs show the posterior probability (PP) values corresponding to *maximum a posteriori* (MAP) states per reconstructed site. The overall accuracy values ($\overline{PP}$) for the reconstructions are: tAncFMO1-5= 0.95, tAncFMO5= 0.97, tAncFMO1-4= 0.94 tAncFMO1-3= 0.95.

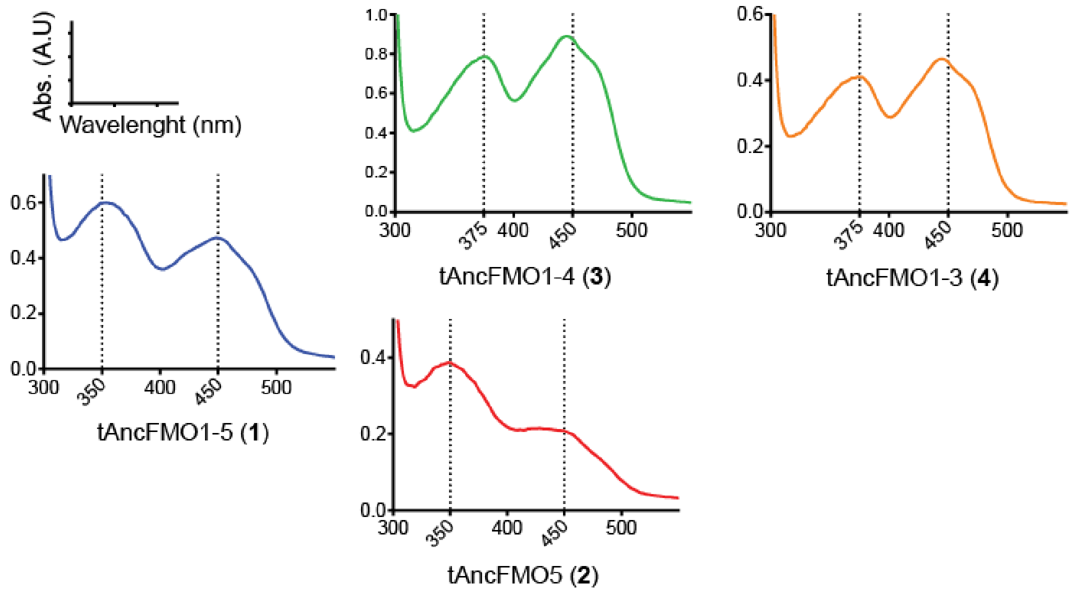

Supplementary Fig. 4

UV-visible spectra of the resurrected FMOs. Spectra were recorded at room temperature after enzyme purification in a Jasco V-660 spectrophotometer.

Enzyme	Melting temperature (T_m) / °C		
	no $NAD(P)^+$	100 equivalents $NADP^+$	100 equivalents NAD^+
**tAncFMO1-5**	60	60	60
**tAncFMO5**	58	57	58
**tAncFMO1-4**	60	60	61
**tAncFMO1-3**	65	65	65

Supplementary Table 1

Melting temperatures of the tAncFMOs. Melting temperatures (T_m), with and without $NAD(P)^+$, were measured in duplicates by the ThermoFAD[3] technique for all tAncFMOs. CFX96 Real-Time PCR System from Bio-Rad was used. Each sample contained 10 µM tAncFMO, in presence or absence of 1 mM $NAD(P)^+$, and topped-up to a final volume of 10 µl with the storage buffer.

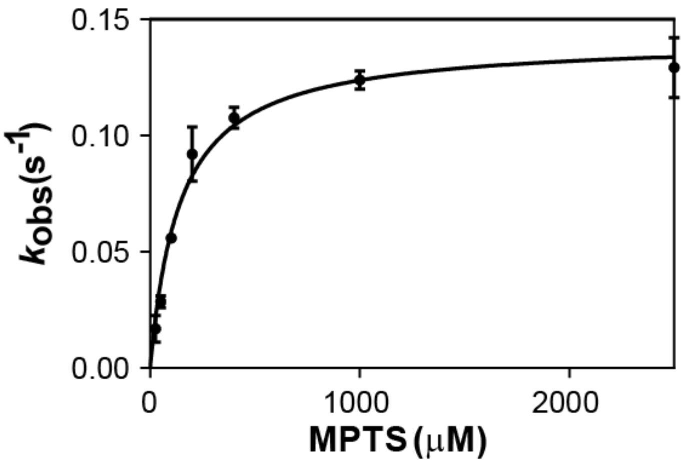

Supplementary Fig. 5

Steady-state kinetics for tAncFMO1-4 towards methyl-*p*-tolyl sulfide (MPTS). NADPH was employed as hydride donor. K_M= 142 ± 16 μM, k_{cat}= 0.14 ± 0.01 s^{-1}. The observed rates were obtained by following the absorbance change at 340 nm. Data are presented as mean values and error bars correspond to SD (n= 3 independent experiments). Source data are provided as a Source Data file.

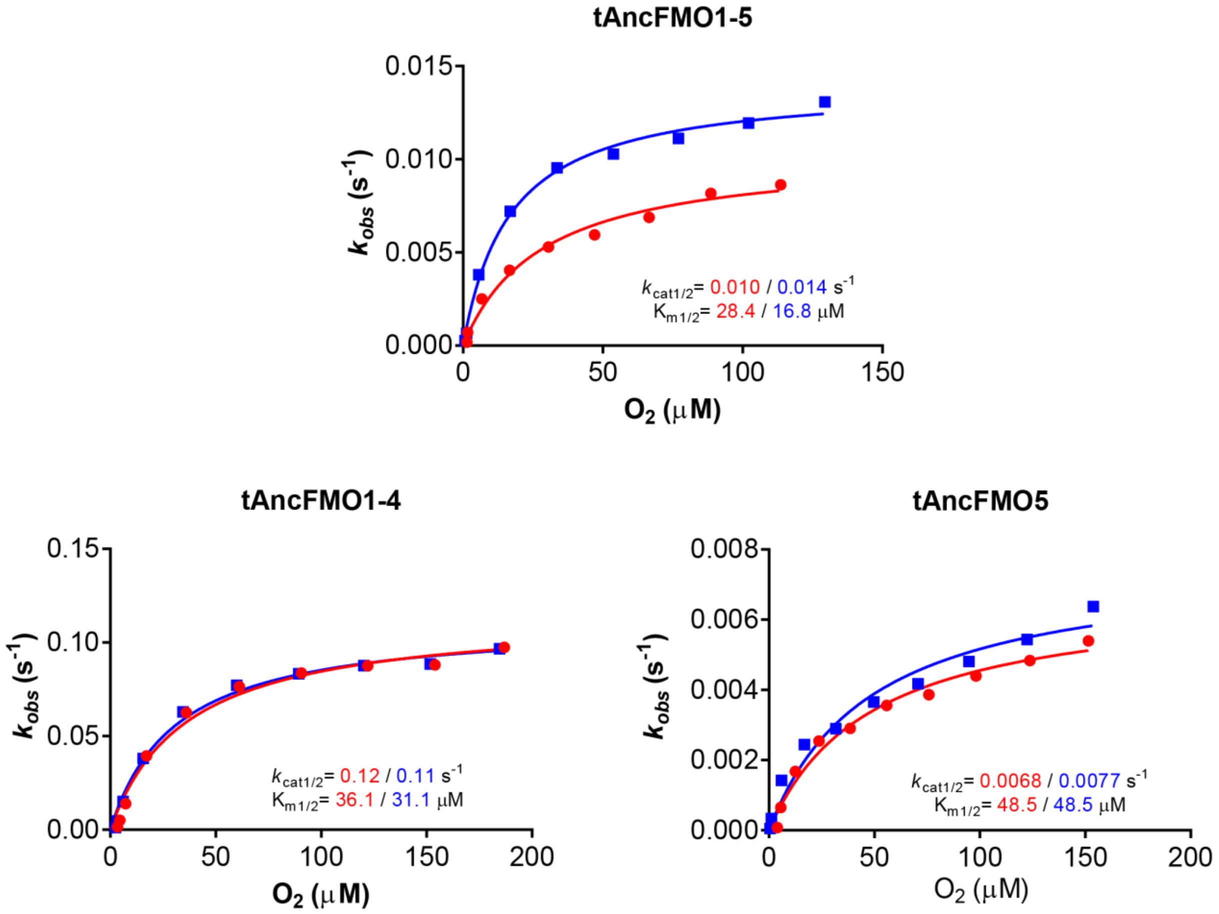

Supplementary Fig. 6

Steady-state kinetics for O_2. 3-12 µM of enzymes were mixed with air-saturated buffer containing excess of NADPH (0.6 mM) and substrate (phenylacetone for tAncFMO5 and methyl-*p*-tolyl sulfide for the other Ancs at 2.5 mM concentration). The k_{obs} was calculated using the OxyTrace^{+} Windows® software. All assays were repeated twice in two single measurements (red and blue traces). Source data are provided as a Source Data file.

Steady-state kinetics					
	**Substrate**	**tAncFMO1-5**	**tAncFMO5**	**tAncFMO1-4**	**tAncFMO1-3**
k_{cat} (s^{-1})	O_2	0.012 ± 0.002	0.0072 ± 0.0005	0.12 ± 0.01	*n.d.*
K_M (μM)		22.6 ± 5.8	48.5 ± 0.1	33.6 ± 2.5	*n.d.*
k_{cat} (s^{-1})	phenylacetone or methyl-*p*-tolyl sulfide	< 0.01	*n.d.*	0.14 ± 0.01	*n.d.*
K_M (μM)		*n.d.*	*n.d.*	142 ± 16	*n.d.*
k_{cat} (s^{-1})	NADPH	*n.d.*	*n.d.*	0.16 ± 0.01	*n.d.*
K_M (μM)		*n.d.*	*n.d.*	15.7 ± 1.8	*n.d.*
k_{cat} (s^{-1})	NADH	*n.d.*	*n.d.*	0.021 ± 0.002	*n.d.*
K_M (μM)		*n.d.*	*n.d.*	90.0 ± 17.6	*n.d.*
k_{unc} (s^{-1})	NADH	0.011 ± 0.001	0.0075 ± 0.0004	0.0061 ± 0.0005	0.0093 ± 0.0006
k_{unc} (s^{-1})	NADPH	0.013 ± 0.001	0.0081 ± 0.0005	0.0067 ± 0.0007	0.019 ± 0.002

Supplementary Table 2

Kinetic parameters of tAncFMOs. Steady-state kinetic parameters are summarized for the characterized tAncFMOs. When O_2 was assayed as substrate, sufficient NADPH and organic substrate (phenylacetone for tAncFMO1-5 & tAncFMO5 and methyl p-tolyl sulfide for tAncFMO1-4 & tAncFMO1-3) were provided. Likewise, when organic substrates were tested, excess of NADPH was employed in the reaction mixtures. When the uncoupling reactions were carried out, no other substrate than the nicotinamide coenzyme was provided in the reaction mixture. Data are presented as mean values ± SD. Source data are provided as a Source Data file.

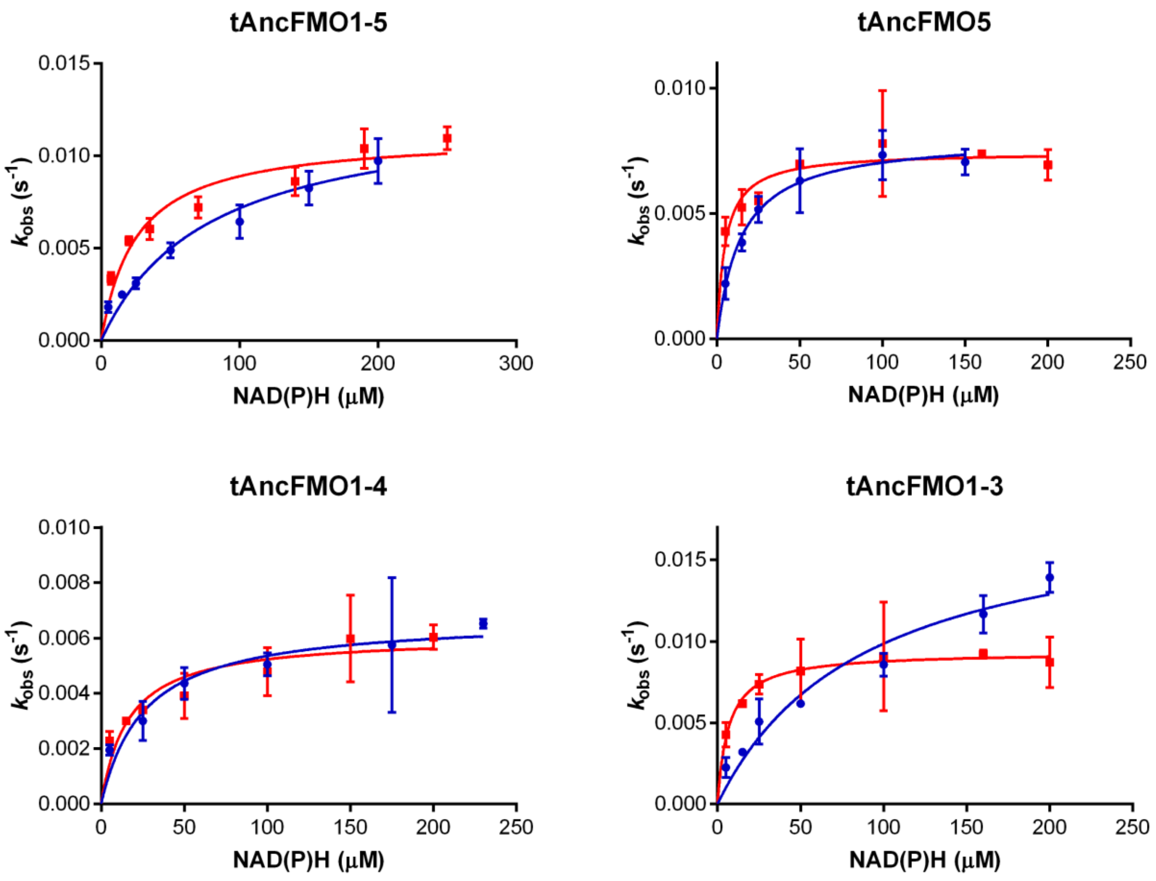

Supplementary Fig. 7

Uncoupling rates measured for tAncFMOs with NADPH (blue) or NADH (red). The rates were measured by following the absorbance change at 340 nm. The reaction mixtures contained 2 or 4 μM of tAncFMOs and NAD(P)H ranging from 5 μM-200 μM. All measurements were carried out in 50 mM KPi buffer containing 250 mM NaCl, 0.05% Triton^{Tm} X-100 reduced and 1% methanol (pH 7.5) at 25 °C. Data are presented as mean values and error bars correspond to SD (n= 3 independent experiments). Source data are provided as a Source Data file.

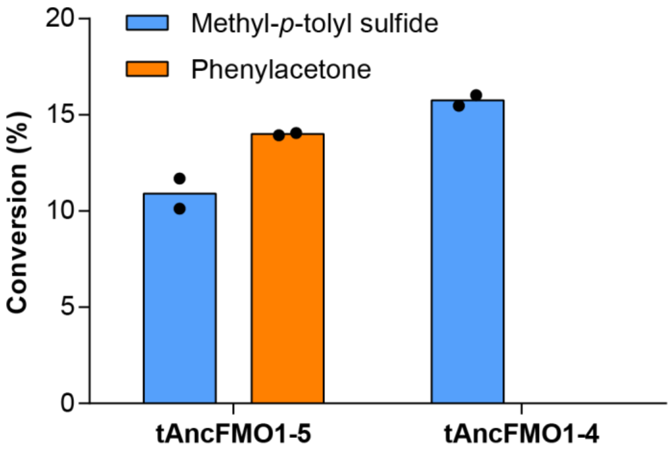

Supplementary Fig. 8

Conversions employing NADH as hydride donor. Two substrates were selected to test the S/N and BV oxidation activities. The reaction mixtures contained 2 µM of enzyme, 2 mM of substrate and 100 µM of NADH. A cofactor recycling system consisting of 5 µM PTDH and 20 mM sodium phosphite was employed to regenerate NADH. All assays were set at 30 °C for 20 h in 50 mM KPi buffer containing 250 mM NaCl, 0.05% Triton^{Tm} X100 and 1% methanol (pH 7.5). Bars represent mean conversion values and dots the values for each experiment (n= 2 independent experiments). Source data are provided in Supplementary Table 4.

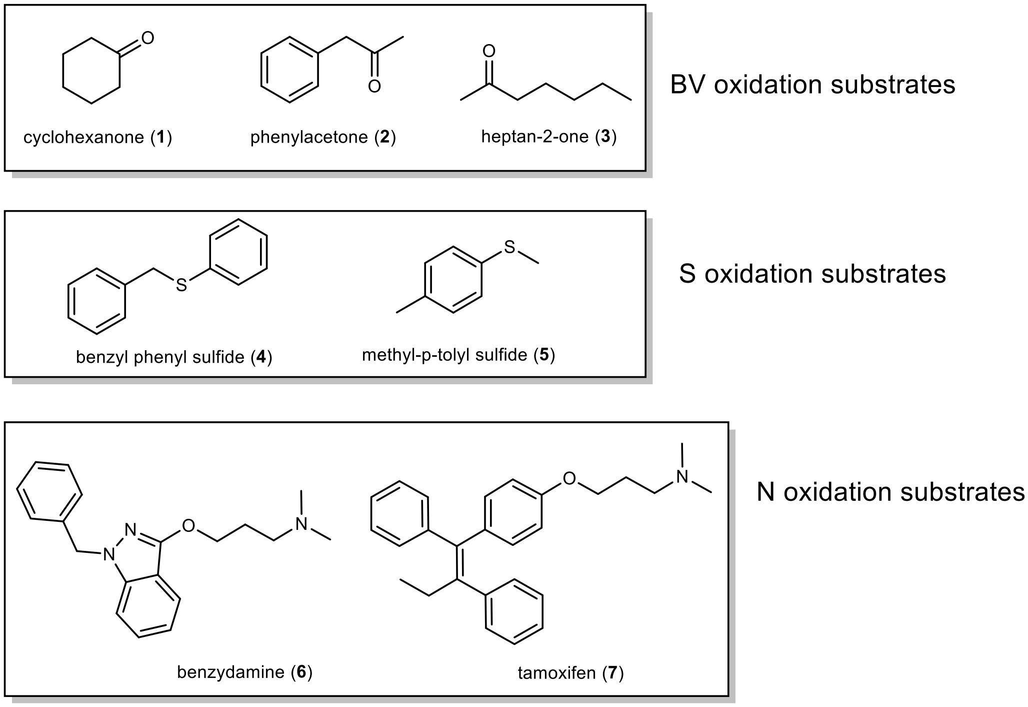

Supplementary Fig. 9

Substrates panel for conversion assays. The BV activity was followed using cyclohexanone, phenylacetone and hepta-2-one as substrates. These were selected for their alicyclic, aromatic and aliphatic features, respectively. Two aromatic thioethers, benzyl phenyl sulfide and methyl-*p*-tolyl sulfide were picked to assess the S oxidation activity. Finally, the N oxidation reactions were determined using bulky and pharmacological relevant molecules as benzydamine and tamoxifen.

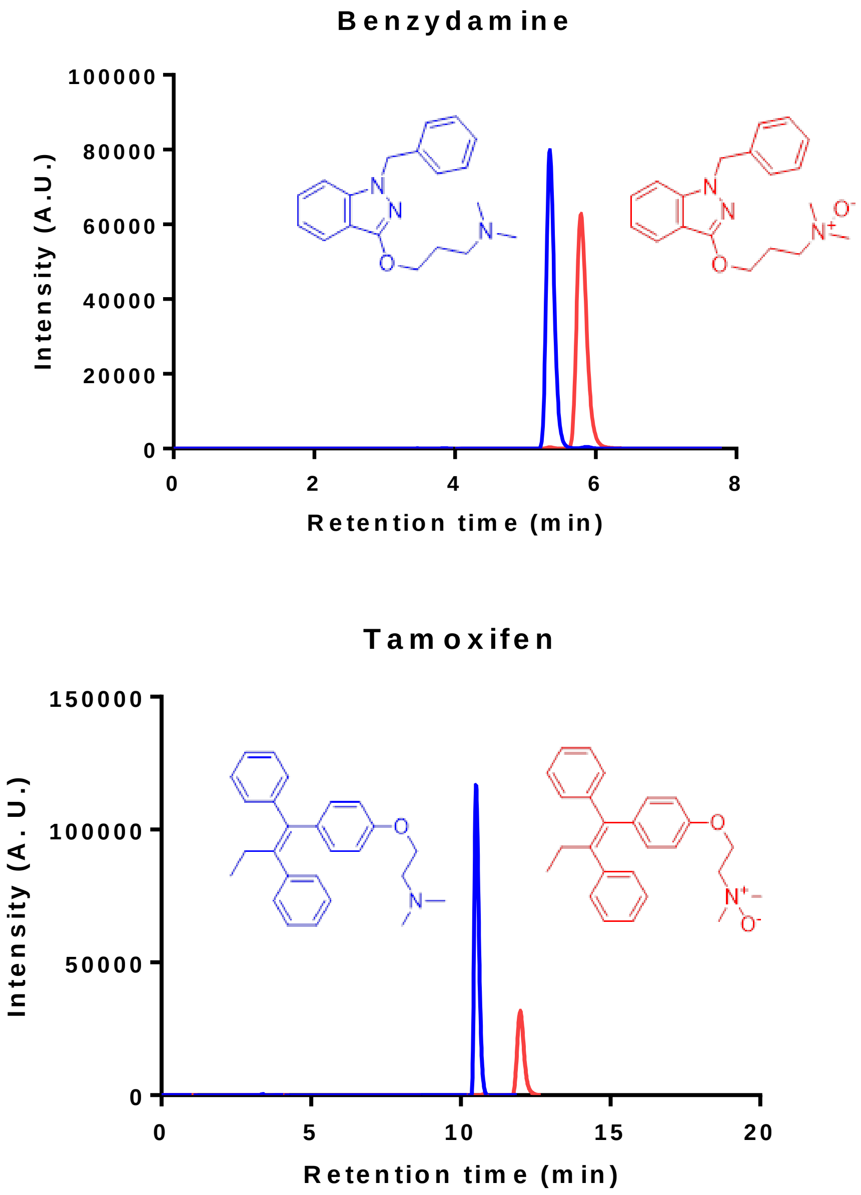

Supplementary Fig. 10

HPLC chromatograms of N oxidation substrates and products. Benzydamine (**6**) and tamoxifen (**7**) peaks are represented in blue and their retention times were 5.3 and 10.5 min, respectively. The benzydamine N-oxide and tamoxifen N-oxide are represented in red with retention times of 5.7 and 11.7 min, respectively. The identity of both products was confirmed employing pure chemical standards.

Substrate		Product	
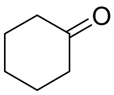<br>Cyclohexanone (**1**)<br>RT: 6.2 min	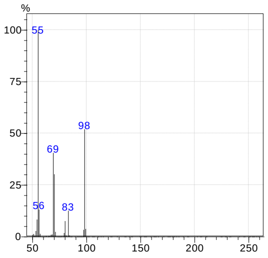	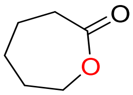<br>RT: 10.7 min	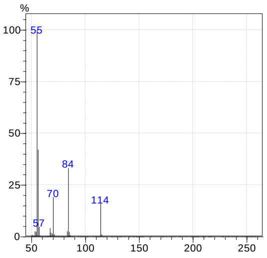
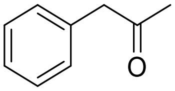<br>Phenylacetone (**2**)<br>RT: 10.9 min	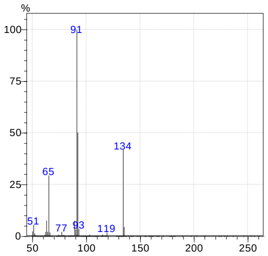	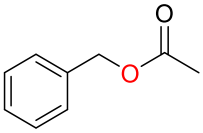<br>RT: 11.6 min	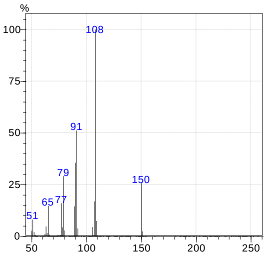
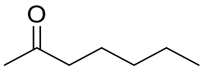<br>Hepta-2-one (**3**)<br>RT: 6.5 min	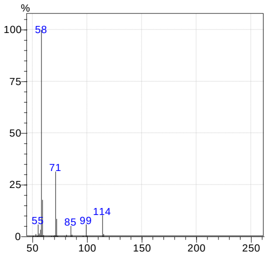	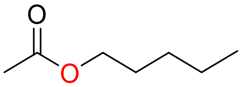<br>RT: 7.1 min	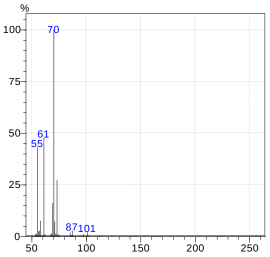
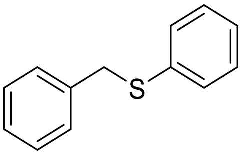<br>Benzyl phenyl sulfide (**4**)<br>RT: 18.8 min	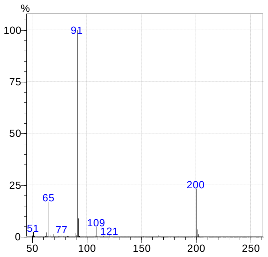	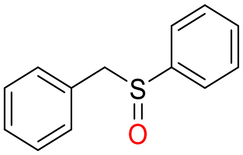<br>RT: 16.7 min	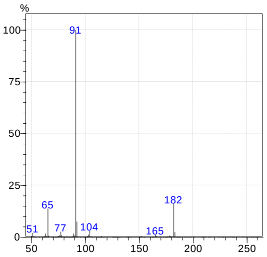
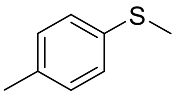<br>Methyl-*p*-tolyl sulfide (**5**)<br>RT: 12.0 min	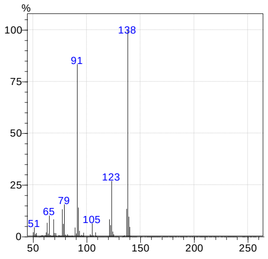	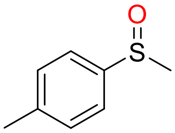<br>RT: 15.5 min	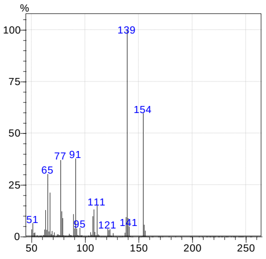

Supplementary Table 3

Selected substrates to evaluate the BV oxidation (1-3) and the S oxidation (4-5) activities. The structures of the expected products on the left. Structures are accompanied by the corresponding MS spectra used to confirm the identity of the analytes. RT: retention times.

Enzyme	BV oxidation						S oxidation				N oxidation			
	Heptan-2-one		Phenylacetone		Cyclohexanone		Benzyl phenyl sulfide		Methyl-*p*-tolyl sulfide		Tamoxifen		Benzydamine	
	R1	R2	R1	R2	R1	R2	R1	R2	R1	R2	R1	R2	R1	R2
tAncFMO1-3	0	0	0	0	0	0	77	78	99	99	48	44	99	99
tAncFMO1-4	0	0	0	0	0	0	53	48	99	99	58	52	44	49
tAncFMO1-5	25	21	23	24	4	6	0	0	32	44	29	31	9	12
tAncFMO5	8	9	17	16	4	6	0	0	37	46	1	1	4	8
mAncFMO2	0	0	0	0	0	0	66	55	57	66	1	1	99	99
mAncFMO1	0	0	0	0	0	0	73	77	62	83	99	92	99	99
mAncFMO3-6	0	0	0	0	0	0	61	51	81	71	27	25	99	99
tAncFMO1-4 | 4x	0	0	0	0	0	0	27	30	37	34	9	8	26	23
tAncFMO1-4 | 4'x	0	0	3	5	0	0	0	0	44	46	14	18	28	25
tAncFMO1-4 | 12x	0	0	3	6	0	0	0	0	20	22	1	1	0	6
tAncFMO1-4 | 16x	0	0	12	6	0	0	0	0	60	49	0	0	7	6
tAncFMO1-4 | N275H	0	0	0	0	0	0	97	95	100	100	89	80	95	92
tAncFMO1-4 | N426H	0	0	0	0	0	0	100	100	100	100	79	63	80	78
tAncFMO1-4 | N275H+N426H	0	0	0	0	0	0	100	100	100	100	59	50	56	49
tAncFMO1-4 | T60I	0	0	0	0	0	0	20	28	100	100	48	54	34	39
tAncFMO1-4 | S222N	0	0	0	0	0	0	38	30	100	100	54	61	40	42
tAncFMO1-4 | T60I+N275H+N426H	3	1	20	14	0	0	32	36	100	100	51	50	40	37
Alt-tAncFMO1-5	58	52	11	12	17	20	33	31	84	87	19	19	6	12
Alt-tAncFMO1-4	0	0	0	0	0	0	93	96	100	100	73	84	51	48

Supplementary Table 4.

tAncFMOs conversion results. The catalytic activities of tetrapods, mammalians, alternative states and mutated tAncFMOs were assessed with the panel of substrates presented in Supplementary Fig. 8. Substrate conversions were done in duplicates using 1.0-5.0 mM substrate (1% methanol), 0.1 mM NADPH, 2.0 µM enzyme, 5.0 µM phosphite dehydrogenase and 20 mM sodium phosphite. All reactions were prepared in 50 mM KPi, 250 mM NaCl, 0.05 % Triton^{Tm} X-100 reduced, pH 7.5. The final reaction volume was adjusted to 1.0 ml and put into 4 ml vials before being incubated at 30 °C with shaking, for 16 h. Conversions of phenylacetone, heptan-2-one, cyclohexanone, benzyl phenyl sulfide and methyl-*p*-tolyl sulfide were analyzed by GC-MS while benzydamine and tamoxifen conversions were monitored by HPLC. Values are given in percentages, with R1 and R2 corresponding to independent experiments (n= 2 independent experiments).

tAncFMO1-5				
Site	MAP state	PP	Alt state	PP
69	Y	0.64	F	0.36
144	L	0.51	M	0.49
152	D	0.54	N	0.42
188	L	0.50	I	0.49
220	I	0.50	V	0.50
234	V	0.69	M	0.21
235	H	0.47	F	0.33
240	K	0.45	Q	0.23
251	L	0.62	M	0.30
256	A	0.69	G	0.24
260	M	0.52	L	0.48
262	Q	0.38	R	0.32
278	F	0.68	L	0.32
339	E	0.75	D	0.25
403	N	0.71	S	0.26
407	A	0.50	E	0.40
415	E	0.58	K	0.38
417	E	0.72	A	0.21
503	V	0.54	A	0.24
507	N	0.76	D	0.23
508	S	0.64	N	0.31
509	S	0.44	T	0.28
511	S	0.59	N	0.34
513	M	0.58	V	0.32
515	L	0.69	F	0.28
519	I	0.71	V	0.21
526	S	0.31	A	0.27
527	A	0.44	S	0.30
528	L	0.35	V	0.27
531	F	0.40	L	0.30
tAncFMO5				
Site	MAP state	PP	Alt state	PP
12	S	0.78	A	0.22
180	Q	0.68	E	0.24
187	V	0.71	I	0.29
251	M	0.65	L	0.32
256	A	0.60	V	0.37
260	M	0.53	L	0.47
350	V	0.69	I	0.31
403	N	0.65	S	0.27
422	T	0.52	K	0.33
503	V	0.59	A	0.26
508	N	0.69	S	0.27
511	S	0.61	N	0.28
513	M	0.62	V	0.30
515	L	0.71	F	0.29
519	I	0.73	V	0.22
526	S	0.28	A	0.25
527	A	0.41	S	0.28
528	L	0.33	V	0.25
531	F	0.37	L	0.29
tAncFMO1-4				
Site	MAP state	PP	Alt state	PP
56	K	0.53	R	0.46
69	Y	0.58	F	0.43
87	F	0.75	L	0.20
91	L	0.74	F	0.26
152	D	0.55	E	0.38

181	E	0.51	G	0.41
192	I	0.66	M	0.27
208	K	0.54	A	0.37
221	L	0.52	M	0.44
236	T	0.49	S	0.43
244	R	0.62	K	0.36
246	I	0.48	V	0.35
251	L	0.47	I	0.42
256	T	0.50	A	0.22
262	Q	0.45	R	0.29
272	V	0.55	I	0.30
277	I	0.50	T	0.23
280	Q	0.70	G	0.26
283	V	0.46	I	0.41
291	R	0.75	C	0.26
293	I	0.74	L	0.26
298	V	0.50	M	0.29
332	S	0.50	T	0.49
339	E	0.73	D	0.27
343	I	0.48	L	0.39
346	E	0.36	Q	0.35
355	Y	0.69	H	0.31
356	V	0.68	I	0.32
382	A	0.54	S	0.26
403	N	0.73	S	0.27
407	A	0.48	E	0.46
416	M	0.72	L	0.20
425	R	0.42	S	0.26
430	T	0.46	V	0.32
442	F	0.54	L	0.22
449	I	0.58	L	0.30
500	K	0.63	R	0.37
503	V	0.44	A	0.21
506	D	0.41	N	0.34
511	N	0.57	S	0.32
513	M	0.41	V	0.40
515	F	0.61	L	0.25
526	S	0.26	A	0.24
527	A	0.40	S	0.27
528	L	0.32	V	0.23
531	F	0.45	L	0.32
**tAncFMO1-3**				
**Site**	**MAP state**	**PP**	**Alt state**	**PP**
56	K	0.53	R	0.46
59	I	0.56	V	0.44
69	Y	0.57	F	0.43
82	M	0.79	L	0.21
152	D	0.53	E	0.45
181	G	0.66	E	0.28
197	A	0.77	S	0.20
208	K	0.54	A	0.37
236	T	0.43	S	0.35
246	V	0.42	I	0.34
277	T	0.63	S	0.29
291	R	0.75	C	0.25
293	I	0.74	L	0.26
298	V	0.50	M	0.39
332	T	0.51	S	0.49
339	D	0.52	E	0.48
355	Y	0.61	H	0.39

356	V	0.66	I	0.34
403	N	0.73	S	0.27
410	A	0.34	N	0.34
449	I	0.71	L	0.26
462	E	0.73	K	0.22
500	R	0.62	K	0.39
506	N	0.33	K	0.22
511	N	0.69	S	0.26
513	V	0.44	I	0.31
526	S	0.23	A	0.22
528	L	0.30	V	0.21
531	F	0.42	L	0.31

Supplementary Table 5.

Ambiguously reconstructed sites in tAncFMOs. Ambiguously reconstructed sites are defined as those for which the second-best state shows a PP > 0.2 (*i.e.:* PP of the MAP state < 0.8).

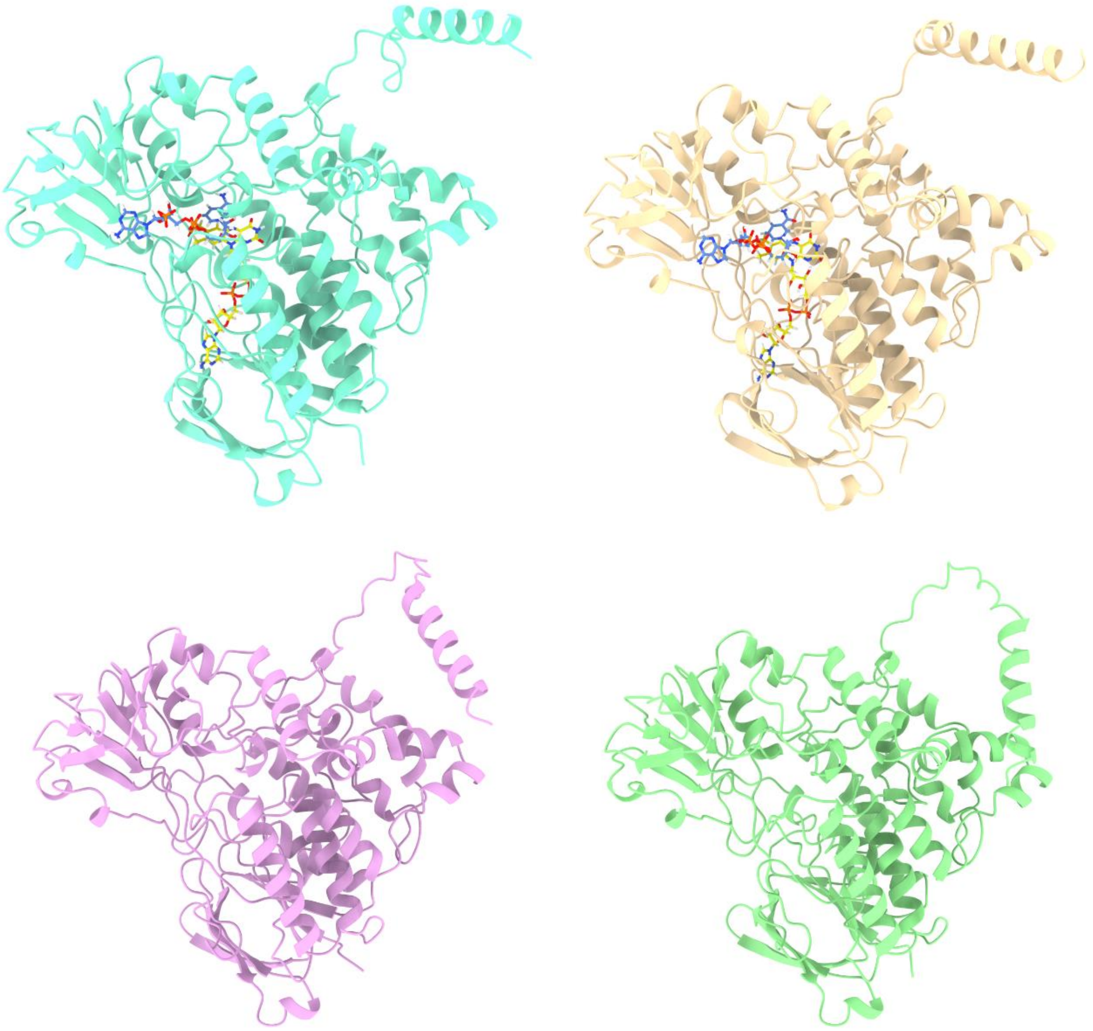

Supplementary Fig. 11

Homology models of tAncFMO1-5 and tAncFMO1-4. Both YASARA (top) and AlphaFold2 (bottom) were used to generate structural models for tAncFMO1-5 and tAncFMO1-4. tAncFMO1-4 is shown in aquamarine and plum, respectively. tAncFMO1-5 is shown in pale orange and pale green, respectively. Molecules of FAD and NADPH are shown in yellow and blue, respectively. As their binding sites are highly conserved among FMOs enzymes, they could be modelled using as references the crystallographically determined structures of the mammalian FMOs. We note that both YASARA and AlphaFold2 models are very similar as outlined by root-mean-square values between their Cα atoms below 1 Å. Images were generated using ChimeraX 1.2.5.

a

tAncFMO1-5 A A K I I D I M F R L D I N H S K K H A R K H
tAncFMO1-4 V S T T F E F L L Q M E V S F T Q R N T W V N
Position 12 17 44 60 74 76 87 88 91|05 44 75|20 22 35 36 40 44 45 56 63 72 75
100 **200**

tAncFMO1-5 F M N S L L Q I S N E E Y H V L L K I N S L
tAncFMO1-4 L V S C V I E T A C K I F N T F I N L D N F
Position 78 83 90 94 98|43 46 81 82 97|15 17 20 26 30 42 49 86 96|07 11 15
300 **400** **500**

b

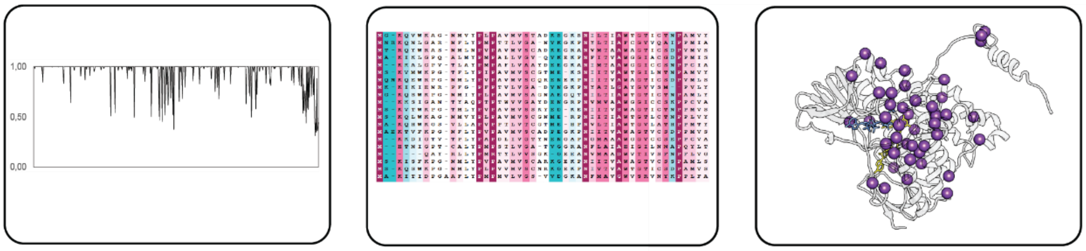

Site	ASR				Degree of conservation			Structural Input
	tAncFMO1-5		tAncFMO1-4		Consurf	BV lineage	S-& N-lineage	
	MAP	PP	MAP	PP				
**12**	A	1.00	V	0.98	4	-	-	Core - FAD-binding domain
**44**	K	1.00	T	1.00	6	-	-	Surface - FAD-binding domain
**60**	I	1.00	T	1.00	**8**	I	T/S	Core - FAD-binding domain
**74**	I	1.00	F	0.94	**7**	I	(variable)	Surface - FAD-binding domain
**222**	N	1.00	S	0.98	**7**	N	S	Surface - 80 residue insertion
**235**	H	0.47	F	0.97	1	-	-	Core - 80 residue insertion
**263**	R	1.00	W	0.98	5	-	-	Surface - 80 residue insertion
**272**	K	0.65	V	0.55	3	-	-	Surface - 80 residue insertion
**275**	H	1.00	N	0.72	**7**	H	(variable)	Surface - 80 residue insertion
**283**	M	0.80	V	0.46	**7**	M/T	V/I	Core - 80 residue insertion
**290**	N	1.00	S	0.98	**8**	N	(variable)	Surface - 80 residue insertion
**294**	S	1.00	C	1.00	**7**	S	(variable)	Surface - 80 residue insertion
**381**	I	1.00	T	0.85	**7**	I	T/G	Core - FAD-binding domain
**417**	E	0.72	I	0.88	2	-	-	Surface - FAD-binding domain
**420**	Y	1.00	F	0.81	**7**	Y	F/G	Surface - FAD-binding domain
**426**	H	1.00	N	0.77	6	-	-	Surface - FAD-binding domain

Supplementary Fig. 12

Reverse mutational strategy. a Alignment of the tAncFMO1-5 and tAncFMO1-4 showing the 45 substitutions occurred at the connecting branch in the phylogeny. **b** The upper scheme depicts the input employed for the integrated analysis: the reconstruction probabilities, the degree of conservation per site and the structural location of the sites shown as pink spheres. The table shows the detailed analysis of the 16 selected sites. The ASR column shows the MAP state of each site and its posterior probabilities in the reconstruction. The degree of conservation was computed by the ConSurf server[4] using the multiple sequence alignment of all the gathered tetrapod sequences. The score ranges from 1 to 9 for variable to conserved sites, respectively. Structural input was based on the previously resolved crystal structures from mAncFMOs and relevant information about the structural position of residues and potential impact on catalysis. The binding domains are assigned as follows: FAD residues 2-154 and 331-442, NADPH residues 155-213 and 296-330, 80 residues insertion 214-295.

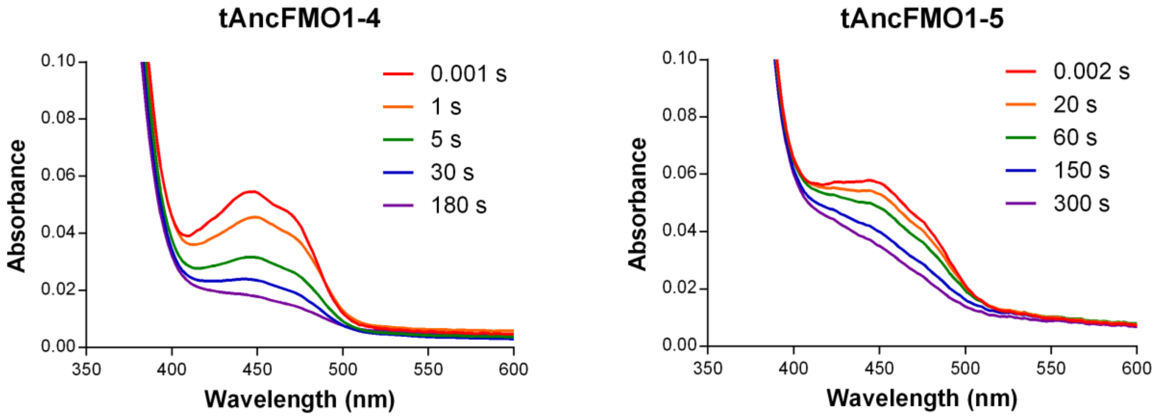

Supplementary Fig. 13

Reduction spectra of tAncFMO1-4 and tAncFMO1-5. On the left, the spectra corresponding to tAncFMO1-4 reduction is shown (k_{red}= 0.25 ± 0.016 s^{-1}). On the right, the spectra corresponding to tAncFMO1-5 (k_{red}= 0.0097 ± 1.2 x10-4 s^{-1}) is shown. Enzyme concentrations were 4 µM and 3.5 µM for tAncFMO1-4 and tAncFMO1-5, respectively. NADPH concentration was 100 µM. All measurements were conducted anaerobically at 25 °C in 50 mM KPi buffer containing 250 mM NaCl and 0.05% *v*/*v* Triton^{Tm} X-100 reduced. All measurements were repeated independently three times (n= 3 independent experiments). Source data are provided as a Source Data file.

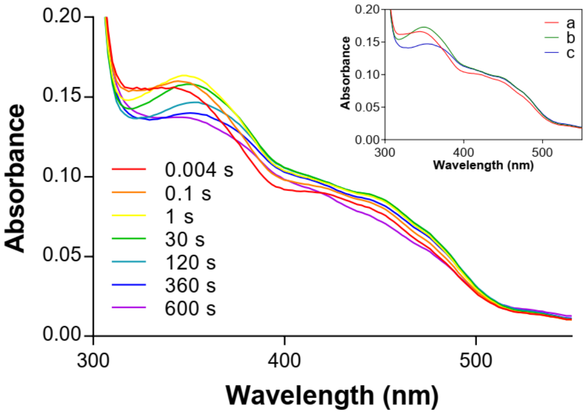

Supplementary Fig. 14

Oxygenating enzyme intermediate formation for tAncFMO1-5. The upper right insets show the deconvoluted spectra fitted into a two-step process $\boldsymbol{a} \xrightarrow{\boldsymbol{k_1}} \boldsymbol{b} \xrightarrow{\boldsymbol{k_2}} \boldsymbol{c}$ with k_1= 1.5 ± 0.1 s^{-1} and k_2= 0.01 ± 0.0005 s^{-1}. Measurements were repeated independently three times (n= 3 independent experiments). Source data are provided as a Source Data file.

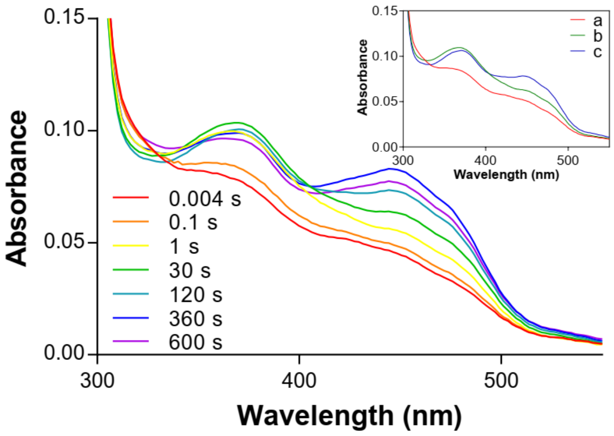

Supplementary Fig. 15

Oxygenating enzyme intermediate formation for 3x-tAncFMO1-4. The upper right insets show the deconvoluted spectra fitted into a two-step process $a \overset{k_1}{\rightarrow} b \overset{k_2}{\rightarrow} c$ with k_1= 1.36 ± 0.43 s^{-1} and k_2= 0.0092 ± 0.0001 s^{-1}. Measurements were repeated independently three times (n= 3 independent experiments). Source data are provided as a Source Data file.

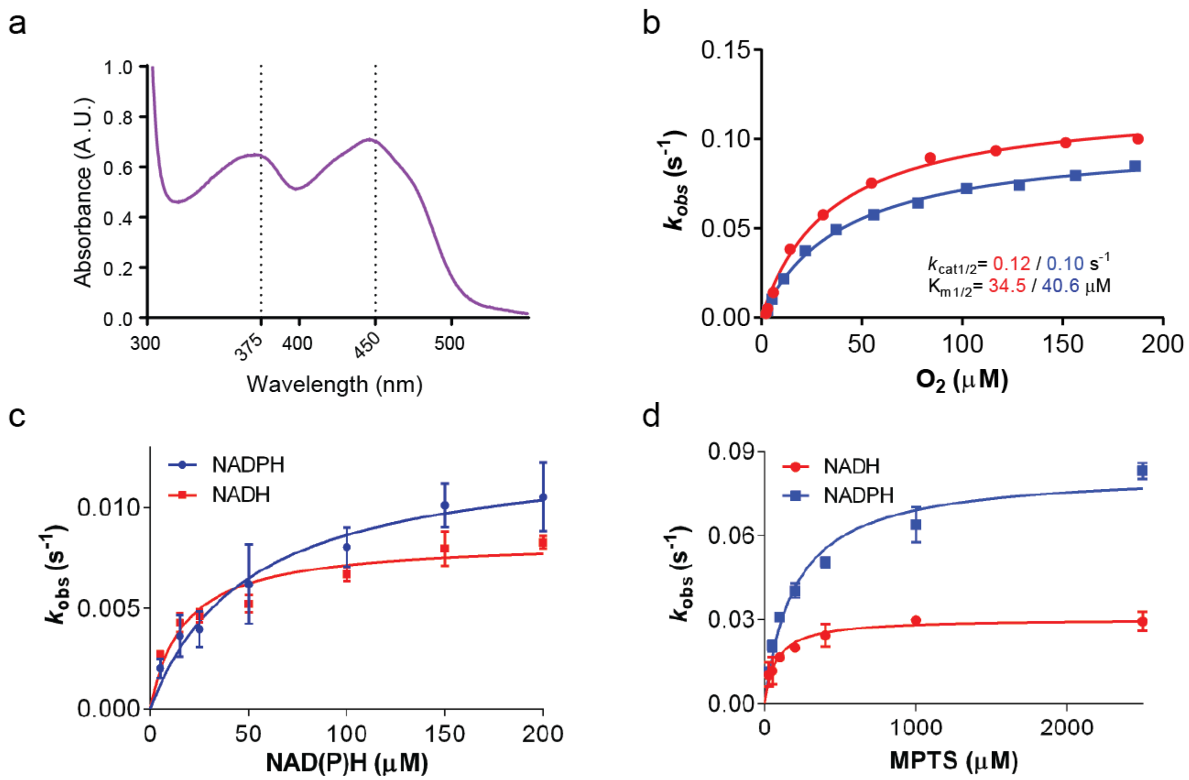

Supplementary Fig. 16

Spectral characterization of the 3x-tAncFMO1-4. a UV-visible spectra of the tAncFMO1-4 triple mutant with dotted lines highlighting the signature absorbance maximums. **b** Steady-state kinetics for O_2. 3 μM of the 3x-tAncFMO1-4 mutant was mixed with air-saturated buffer containing excess of NADPH (0.6 mM) and methyl-*p*-tolyl sulfide (2.5 mM). The k_{obs} at a certain time was calculated by OxyTrace^{+} Windows® software. All assays were repeated twice (red and blue). **c** Uncoupling rates measured employing NADPH (blue) or NADH (red). The observed rates in the graph were obtained by following the absorbance change at 340 nm. The reaction mixtures contained 2 μM of enzyme and a variable NAD(P)H concentration from 5 μM-200 μM. All measurements were carried out in 50 mM KPi buffer containing 250 mM NaCl, 0.05% Triton^{Tm} X-100 reduced and 1% methanol (pH 7.5) at 25 °C. Data are presented as mean values and error bars correspond to SD (n= 3 independent experiments). **d** Steady-state kinetics towards methyl-*p*-tolyl sulfide (MPTS). The reaction mixtures contained 2 μM of enzyme, 100 μM NADPH and a variable MPTS concentration from 25 μM to 2500 μM. All measurements were carried out in 50 mM KPi buffer containing 250 mM NaCl, 0.05% Triton^{Tm} X-100 reduced and 1% methanol (pH 7.5) at 25 °C. Data are presented as mean values and error bars correspond to SD (n= 3 independent experiments). Source data are provided as a Source Data file.

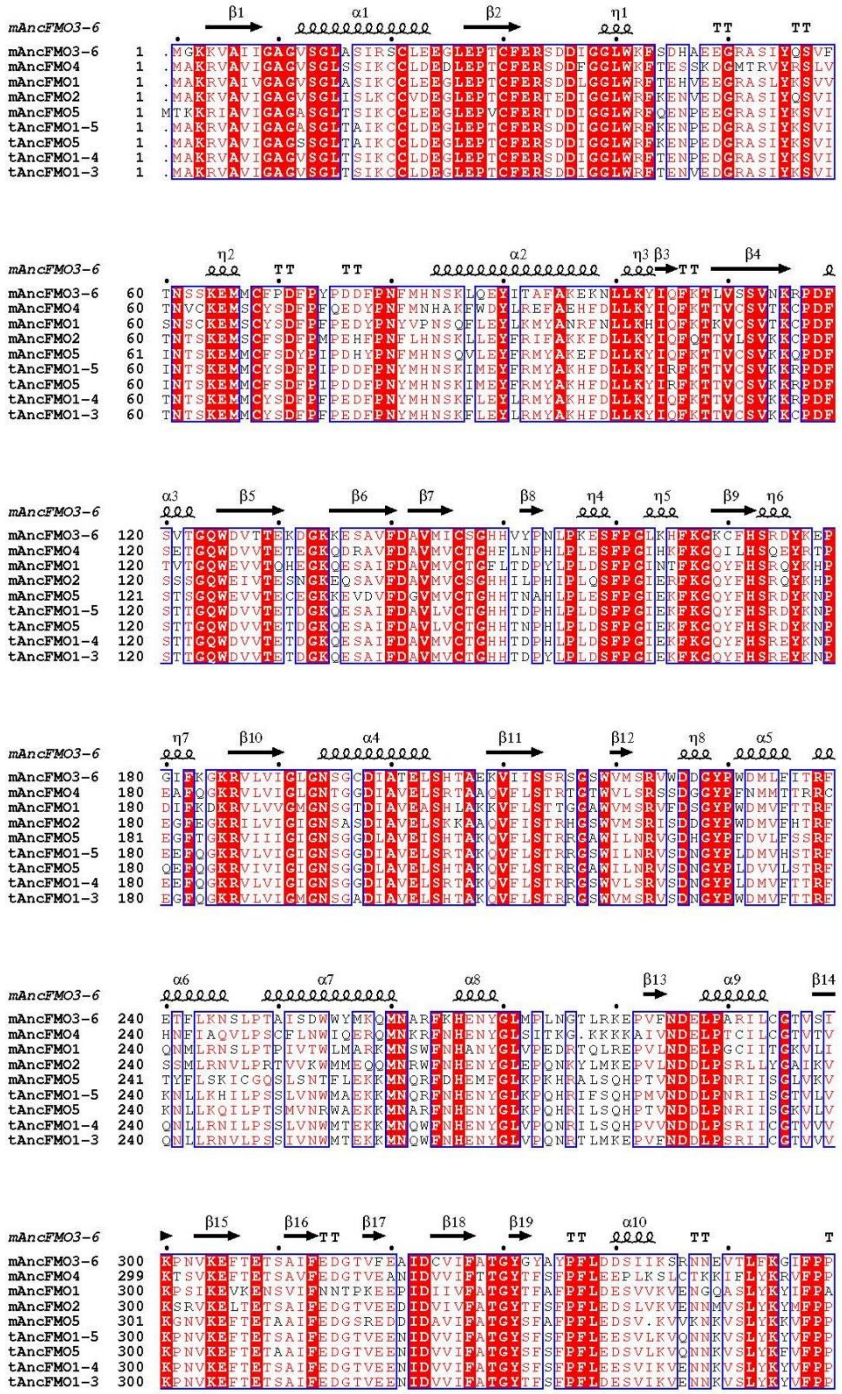
mAncFMO3-6
mAncFMO4
mAncFMO1
mAncFMO2
mAncFMO5
tAncFMO1-5
tAncFMO5
tAncFMO1-4
tAncFMO1-3

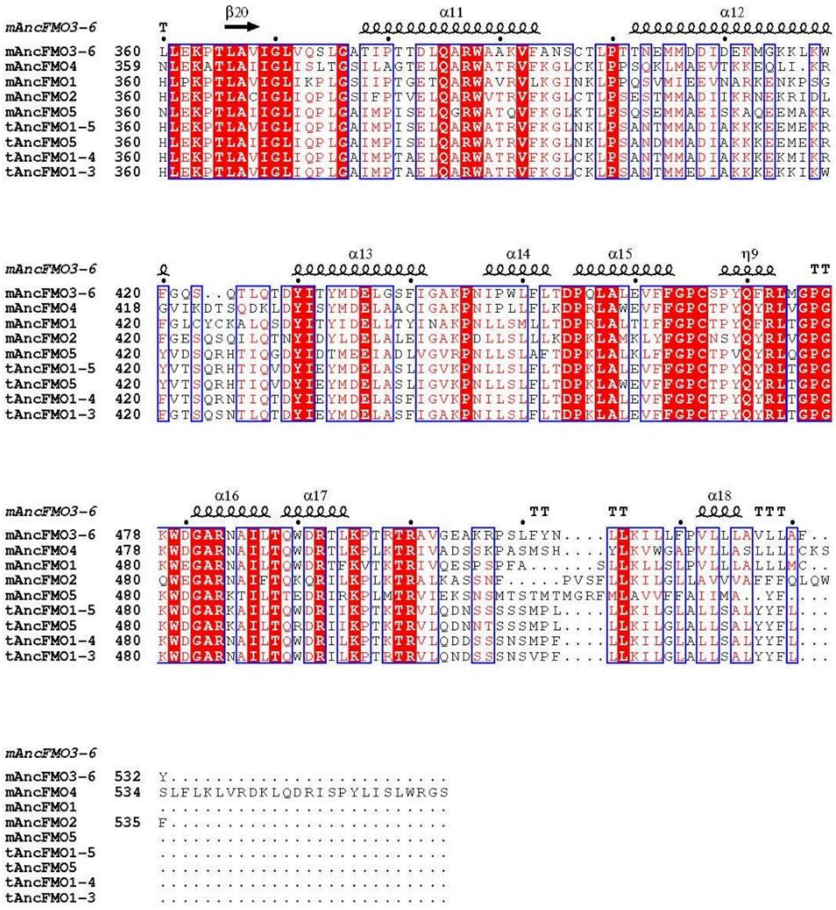

Supplementary Fig. 17

Multiple sequence alignment (MSA) of tAncFMOs. The MSA was obtained using the ENDscript server (https://endscript.ibcp.fr)[5] and the structural features of mAncFMO3-6 are given as reference.

SUPPLEMENTARY REFERENCES

1. Bailleul, G., Nicoll, C.R., Mascotti, M.L., Mattevi, A. & Fraaije, M.W. Ancestral reconstruction of mammalian FMO1 enables structural determination, revealing unique features that explain its catalytic properties. *Journal of Biological Chemistry* **296**, 100221 (2021).
2. Nicoll, C.R. et al. Ancestral-sequence reconstruction unveils the structural basis of function in mammalian FMOs. *Nature Structural & Molecular Biology* **27**, 14-24 (2020).
3. Forneris, F., Orru, R., Bonivento, D., Chiarelli, L.R. & Mattevi, A. ThermoFAD, a Thermofluor((R))-adapted flavin ad hoc detection system for protein folding and ligand binding. *Febs Journal* **276**, 2833-2840 (2009).
4. Ashkenazy, H. et al. ConSurf 2016: an improved methodology to estimate and visualize evolutionary conservation in macromolecules. *Nucleic Acids Research* **44**, W344-W350 (2016).
5. Robert, X. & Gouet, P. Deciphering key features in protein structures with the new ENDscript server. *Nucleic Acids Research* **42**, W320-W324 (2014).
