# Supplementary material for: Evolution of enzyme functionality in the flavin-containing monooxygenases
Source: Nat Commun. 2023 Feb 24;14:1042. doi: 10.1038/s41467-023-36756-x (PMC9950137; doi:10.1038/s41467-023-36756-x)
Supplement: Supplementary file 3 — Description of additional supplementary files [file 41467_2023_36756_MOESM3_ESM.docx]

**Supplementary Data 1**. Multiple sequence alignment (MSA) of the collected FMOs in fasta format (536 sequences, 537 sites).
