# Supplementary material for: Evolution of enzyme functionality in the flavin-containing monooxygenases
Source: Nat Commun. 2023 Feb 24;14:1042. doi: 10.1038/s41467-023-36756-x (PMC9950137; doi:10.1038/s41467-023-36756-x)
Supplement: Supplementary file 5 — Reporting Summary [file 41467_2023_36756_MOESM5_ESM.pdf]

nature portfolio

Corresponding author(s): Maria Laura Mascotti

Last updated by author(s): Jan 24, 2023

Reporting Summary

Nature Portfolio wishes to improve the reproducibility of the work that we publish. This form provides structure for consistency and transparency in reporting. For further information on Nature Portfolio policies, see our Editorial Policies and the Editorial Policy Checklist.

Statistics

For all statistical analyses, confirm that the following items are present in the figure legend, table legend, main text, or Methods section.

n/a	Confirmed	
☐	☒	The exact sample size (*n*) for each experimental group/condition, given as a discrete number and unit of measurement
☐	☒	A statement on whether measurements were taken from distinct samples or whether the same sample was measured repeatedly
☒	☐	The statistical test(s) used AND whether they are one- or two-sided<br>*Only common tests should be described solely by name; describe more complex techniques in the Methods section.*
☒	☐	A description of all covariates tested
☒	☐	A description of any assumptions or corrections, such as tests of normality and adjustment for multiple comparisons
☐	☒	A full description of the statistical parameters including central tendency (e.g. means) or other basic estimates (e.g. regression coefficient) AND variation (e.g. standard deviation) or associated estimates of uncertainty (e.g. confidence intervals)
☒	☐	For null hypothesis testing, the test statistic (e.g. *F*, *t*, *r*) with confidence intervals, effect sizes, degrees of freedom and *P* value noted<br>*Give P values as exact values whenever suitable.*
☐	☒	For Bayesian analysis, information on the choice of priors and Markov chain Monte Carlo settings
☒	☐	For hierarchical and complex designs, identification of the appropriate level for tests and full reporting of outcomes
☒	☐	Estimates of effect sizes (e.g. Cohen's *d*, Pearson's *r*), indicating how they were calculated

Our web collection on statistics for biologists contains articles on many of the points above.

Software and code

Policy information about availability of computer code

Data collection	Sequences were collected from NCBI non-redundant protein sequences (nr) and nucleotide collection (nr/nt), and UniprotKB. BLASTP, tBLASTn and HMMER searching algorithms were employed. Species evolutionary history was retrieved from TimeTree (http://www.timetree.org/).
Data analysis	The following software were employed: MAFFT v7,RAXML v8.2.10, Mr. Bayes v3.2.6, PROTTEST v3.4, BOOSTER (https://booster.pasteur.fr/), Figtree v1.4.2, PAML 4.9a, Jalview v2.11.2.6, MEGA x, AlphaFold2, YASARA 22.9.24, Chimera X v1.2.5, Pymol v2.5.2, ConSurf (https://consurf.tau.ac.il/consurf_index.php), Pro-Data Viewer v4.2.12, Pro-Kineticist 1.0.13, GraphPad Prism 6.05 and OxyTrace+ v1.0 build 48.

For manuscripts utilizing custom algorithms or software that are central to the research but not yet described in published literature, software must be made available to editors and reviewers. We strongly encourage code deposition in a community repository (e.g. GitHub). See the Nature Portfolio guidelines for submitting code & software for further information.

Data

Policy information about availability of data

All manuscripts must include a data availability statement. This statement should provide the following information, where applicable:

- Accession codes, unique identifiers, or web links for publicly available datasets
- A description of any restrictions on data availability
- For clinical datasets or third party data, please ensure that the statement adheres to our policy

DATA AVAILABILITY

The ancestral sequences (tAncFMOs) generated in this study have been deposited in the Genbank database under accession codes: OP381052 [https://www.ncbi.nlm.nih.gov/nuccore/OP381052.1/] (tAncFMO1-5), OP381053 [https://www.ncbi.nlm.nih.gov/nuccore/OP381053](tAncFMO5), OP381054 [https://www.ncbi.nlm.nih.gov/nuccore/OP381054] (tAncFMO1-4) and OP381055 [https://www.ncbi.nlm.nih.gov/nuccore/OP381055] (tAncFMO1-3). The experimental data generated in this study is provided in the Supplementary Information/Source Data file. The collected dataset for the phylogenetic analysis is provided in the Supplementary Information. The taxonomic relationships and evolutionary timescale data used in this study are available in the TimeTree 5 knowledge-base [http://www.timetree.org/]. The silhouette images of organisms used in this study are available in the PhyloPic database [http://www.phylopic.org/]. The structural data used in this study are available in the PDB database under accession codes: 6SEK [10.2210/pdb6SF0/pdb] (mAncFMO5) and 6SF0 [10.2210/pdb6SF0/pdb] (mAncFMO2). The sequence data used in this study are available in the Genbank database under accession codes: OP381050 [https://www.ncbi.nlm.nih.gov/nuccore/2366301618] (mAncFMO1), OP381047 [https://www.ncbi.nlm.nih.gov/nuccore/2366301433] (mAncFMO2), OP381048 [https://www.ncbi.nlm.nih.gov/nuccore/2366301494] (mAncFMO3-6) and OP381049 [https://www.ncbi.nlm.nih.gov/nuccore/2366301561] (mAncFMO5).

Human research participants

Policy information about studies involving human research participants and Sex and Gender in Research.

Reporting on sex and gender	N/A
Population characteristics	N/A
Recruitment	N/A
Ethics oversight	N/A

Note that full information on the approval of the study protocol must also be provided in the manuscript.

Field-specific reporting

Please select the one below that is the best fit for your research. If you are not sure, read the appropriate sections before making your selection.

[x] Life sciences [ ] Behavioural & social sciences [ ] Ecological, evolutionary & environmental sciences

For a reference copy of the document with all sections, see nature.com/documents/nr-reporting-summary-flat.pdf

Life sciences study design

All studies must disclose on these points even when the disclosure is negative.

Sample size	For steady-state and pre steady-state kinetic measurements typically n= 3 independent experiments. For each experiment the number of measurements (variable substrate concentrations, i.e.: points) was initially set between 6-8. Further points were assayed to obtain precise kinetic parameters.<br>Conversion assays were done in duplicates (two independent measurements).<br>The n employed for each measurement is the standard for enzyme kinetics determinations/biocatalysis assays.
Data exclusions	No data were excluded from the analyses
Replication	Kinetic measurements were performed three independent times. Conversions were done in two different experiments, if measurements resulted in highly dissimilar values (>10% difference among both), further replicates were performed. Fast kinetic experiments were repeated at least three independent times (typically five times and the first two measurements are discarded).<br>All attempts of replication were successful, leading to the calculation of consistent kinetic parameters.
Randomization	Randomization is not typically applied in enzyme kinetics determinations/biocatalysis assays.
Blinding	Blinding is not typically applied in enzyme kinetics determinations/biocatalysis assays.

Reporting for specific materials, systems and methods

We require information from authors about some types of materials, experimental systems and methods used in many studies. Here, indicate whether each material, system or method listed is relevant to your study. If you are not sure if a list item applies to your research, read the appropriate section before selecting a response.

Materials & experimental systems

n/a	Involved in the study
☒	☐ Antibodies
☒	☐ Eukaryotic cell lines
☒	☐ Palaeontology and archaeology
☒	☐ Animals and other organisms
☒	☐ Clinical data
☒	☐ Dual use research of concern

Methods

n/a	Involved in the study
☒	☐ ChIP-seq
☒	☐ Flow cytometry
☒	☐ MRI-based neuroimaging
